# Proteomics approach combined with biochemical attributes to elucidate compatible and incompatible plant-virus interactions between *Vigna mungo* and Mungbean Yellow Mosaic India Virus

**DOI:** 10.1186/1477-5956-11-15

**Published:** 2013-04-15

**Authors:** Subrata Kundu, Dipjyoti Chakraborty, Anirban Kundu, Amita Pal

**Affiliations:** 1 Division of Plant Biology, Bose Institute, Kolkata, WB, 700054, India; 2 Department of Bioscience and Biotechnology, Banasthali Vidyapith, Rajasthan, 304022, India

**Keywords:** Incompatible interaction, Compatible interaction, Mungbean Yellow Mosaic India Virus, *Vigna mungo*, 2-dimensional gel electrophoresis, MALDI-TOF/TOF

## Abstract

**Background:**

*Vigna mungo*, a tropical leguminous plant, highly susceptible to yellow mosaic disease caused by Mungbean Yellow Mosaic India Virus (MYMIV) resulting in high yield penalty. The molecular events occurring during compatible and incompatible interactions between *V*. *mungo* and MYMIV pathosystem are yet to be explored. In this study biochemical analyses in conjunction with proteomics of MYMIV-susceptible and -resistant *V. mungo* genotypes were executed to get an insight in the molecular events during compatible and incompatible plant-virus interactions.

**Results:**

Biochemical analysis revealed an increase in phenolics, hydrogen peroxide and carbohydrate contents in both compatible and incompatible interactions; but the magnitudes were higher during incompatible interaction. In the resistant genotype the activities of superoxide dismutase and ascorbate peroxidase increased significantly, while catalase activity decreased. Comparative proteome analyses using two-dimensional gel electrophoresis coupled with mass spectrometry identified 109 differentially abundant proteins at 3, 7 and 14 days post MYMIV-inoculation. Proteins of several functional categories were differentially changed in abundance during both compatible and incompatible interactions. Among these, photosynthesis related proteins were mostly affected in the susceptible genotype resulting in reduced photosynthesis rate under MYMIV-stress. Differential intensities of chlorophyll fluorescence and chlorophyll contents are in congruence with proteomics data. It was revealed that Photosystem II electron transports are the primary targets of MYMIV during pathogenesis. Quantitative real time PCR analyses of selected genes corroborates with respective protein abundance during incompatible interaction. The network of various cellular pathways that are involved in inducing defense response contains several conglomerated cores of nodal proteins, of which ascorbate peroxidase, rubisco activase and serine/glycine hydroxymethyl transferase are the three major hubs with high connectivity. These nodal proteins play the crucial role of key regulators in bringing about a coordinated defense response in highly orchestrated manner.

**Conclusions:**

Biochemical and proteomic analyses revealed early accumulation of the defense/stress related proteins involved in ROS metabolism during incompatible interaction. The robustness in induction of defense/stress and signal transduction related proteins is the key factor in inducing resistance. The mechanism of MYMIV-resistance in *V. mungo* involves redirection of carbohydrate flux towards pentose phosphate pathway. Some of these identified, differentially regulated proteins are also conferring abiotic stress responses illustrating harmony amongst different stress responses. To the best of our knowledge, this is the lone study deciphering differential regulations of *V*. *mungo* leaf proteome upon MYMIV infection elucidating the mode of resistance response at the biochemical level.

## Background

Plants exhibit specific responses when challenged with viruses. Compatible host-virus interactions result in systemic infections leading to the development of characteristic symptoms. The magnitude of physiological and phenotypic changes in the host during viral infection suggests the activation and suppression of global gene expressions in the host [1]. In incompatible interaction, the expression of host resistance (R) gene is triggered by specific molecular interactions with viral avirulence (Avr) proteins and activates a cascade of genes to induce defense mechanisms including synthesis of pathogenesis-related (PR) proteins [2,3]. The accumulation of PR proteins is also associated with systemic acquired resistance (SAR) against a wide range of pathogens [4]. During incompatible interaction the virus replication is ceased and the movement is arrested at or near the sites of infection. The ‘oxidative burst’ by the production of reactive oxygen species (ROS) is one of the earliest cellular responses following pathogen infection. The sequential reduction of molecular oxygen to superoxide radical (.O_2_^-^), hydrogen peroxide (H_2_O_2_) and hydroxyl radical (.OH) are the most predominant ROS produced in plant cell. ROS-scavenging enzymes, including ascorbate peroxidases (APX), superoxide dismutases (SOD) and catalase (CAT) maintain ROS homeostasis in different compartments of the plant cell [5]. The ROS either destroy the invading pathogens directly or activate expression of defense related gene cascade [6].

In this communication we report interactions between *V. mungo* and a viral pathogen, MYMIV at the biochemical and physiological level. MYMIV belongs to the genus begomovirus and causes yellow mosaic disease (YMD) in several edible grain legumes including *V. mungo, V. radiata, Glycine max, Phaseolus angularis, P. lunatus* and *P. vulgaris*. YMD spreads all over the South-Asian countries leading to a maximum of cent percent yield penalty when infestation occurs at the juvenile developmental stage. The disease is transmitted by Whitefly (*Bemisia tabaci*) that delivers the virus through proboscis to phloem cells of the host plant. Consequently, the virus propagates within the susceptible plants using host’s cellular machinery and causes irregular chlorotic yellow patches on the leaf lamina, which gradually covers the entire surface and leaves become yellow [7,8]. However, the molecular events occurring in susceptible and resistant host plants after virus invasions are yet to be deciphered. Considering the importance of *V. mungo* as an important pulse crop, host proteins that modulate during virus invasion were identified to elucidate their roles in regulating the resistance response. Moreover, there is limited information on physiological and biochemical responses of *V. mungo* upon MYMIV infection. Therefore, the present study was extended to investigate differences in physiological and biochemical parameters between MYMIV-susceptible and -resistant genotypes at different time points following whitefly mediated inoculation of MYMIV.

## Results and discussion

### Biochemical changes in *V. mungo* during compatible and incompatible interactions

Appearance of disease symptoms in leaves of *V. mungo* during compatible interaction was noted after artificial inoculation with MYMIV, as mentioned in Materials and Methods. No such symptom development was observed during incompatible interaction in the MYMIV-resistant plants after challenging with the virus. Symptomatic changes in leaf morphology during compatible interaction at different time points are shown in Additional file 1: Figure S1. The susceptible genotype inoculated with MYMIV showed YMD symptoms in the form of yellow patches. In contrast, leaves of the resistant genotype were devoid of any chlorotic tissue showing resistance response against invading virus. The observation was corroborated with the presence of MYMIV coat protein DNA fragment (GenBank ID. HQ221570) in the susceptible genotype at 14 dpi (Additional file 1: Figure S1B). On the other hand, due to low level of virus titer in the infected leaf tissues of MYMIV-resistant genotype only a faint amplified product could be seen after repeated re-PCR (at least four times) of the PCR-amplified-product.

**Figure 1:**
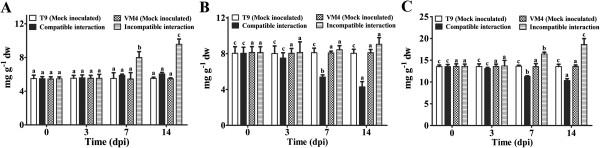
**Effect of MYMIV inoculation on soluble, insoluble and total carbohydrate content of *Vigna mungo *leaves.** (**A**) insoluble (**B**) and total carbohydrate (**C**) content of *V.mungo* leaves. Bars indicate mean ± SD. The ANOVA and DMRT were performed between mock inoculated T9 and compatible interaction; and mock inoculated VM4 vs. incompatible interaction. Bars followed by same alphabets are not significantly different at p ≤ 0.05.

During incompatible interaction soluble and total carbohydrate content increased significantly at 7 and 14 dpi (Figure 1A and C); and a marginal increase in the amount of soluble carbohydrate in compatible interaction has been noted at 7 and 14 dpi (Figure 1B). Whereas the insoluble carbohydrate content increased slightly in resistant genotype at 14 dpi but drastically reduced in the infected susceptible plants at 7 and 14 dpi (Figure 1B). Changes in the carbohydrate contents in mock inoculated plants of both the genotypes were negligible (Figure 1A-C).

**Figure 2:**
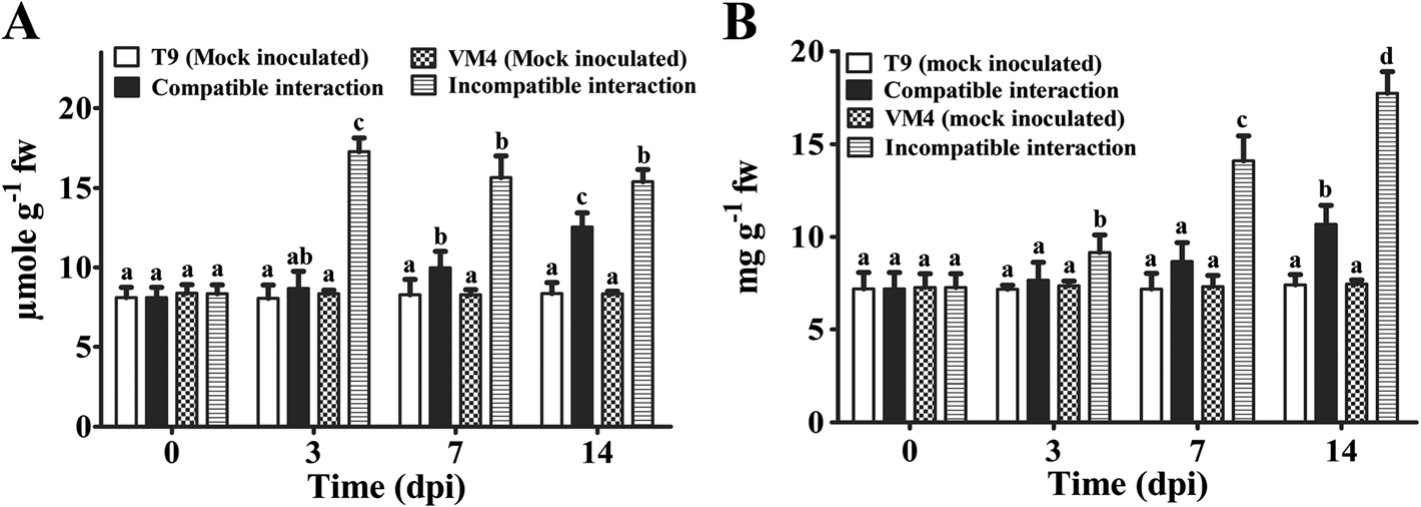
**Effect of MYMIV inoculation on H_2_O_2_ and total phenolic content of *Vigna mungo *leaves.** (**A**) and total phenolic (**B**) content of *V*. *mungo* leaves. Bars indicate mean ± SD. The ANOVA and DMRT were performed between mock inoculated T9 and compatible interaction; and mock inoculated VM4 vs. incompatible interaction. Bars followed by same alphabets are not significantly different at p ≤ 0.05.

An increase in H_2_O_2_ and total phenolic content was observed in both types of interactions but the magnitude of induction was much higher during incompatible interaction (Figure 2A, B). The H_2_O_2_ level abruptly increased to 2-fold in resistant genotype at 3 dpi (Figure 2A). A maximum of 2.4-fold increase in phenolic content was observed in resistant genotype at 14 dpi (Figure 2B). In mock inoculated plants, H_2_O_2_ and total phenolic content did not change.

A significant decrease in Chlorophyll a (chl a), Chlorophyll b (chl b) and in carotenoid content was observed during compatible interaction at 7 and 14 dpi (Additional file 2: Figure S2A-C). These parameters did not change significantly during incompatible interaction. The chl a, chl b and carotenoid content were comparable in two mock inoculated genotypes.

**Figure 3:**
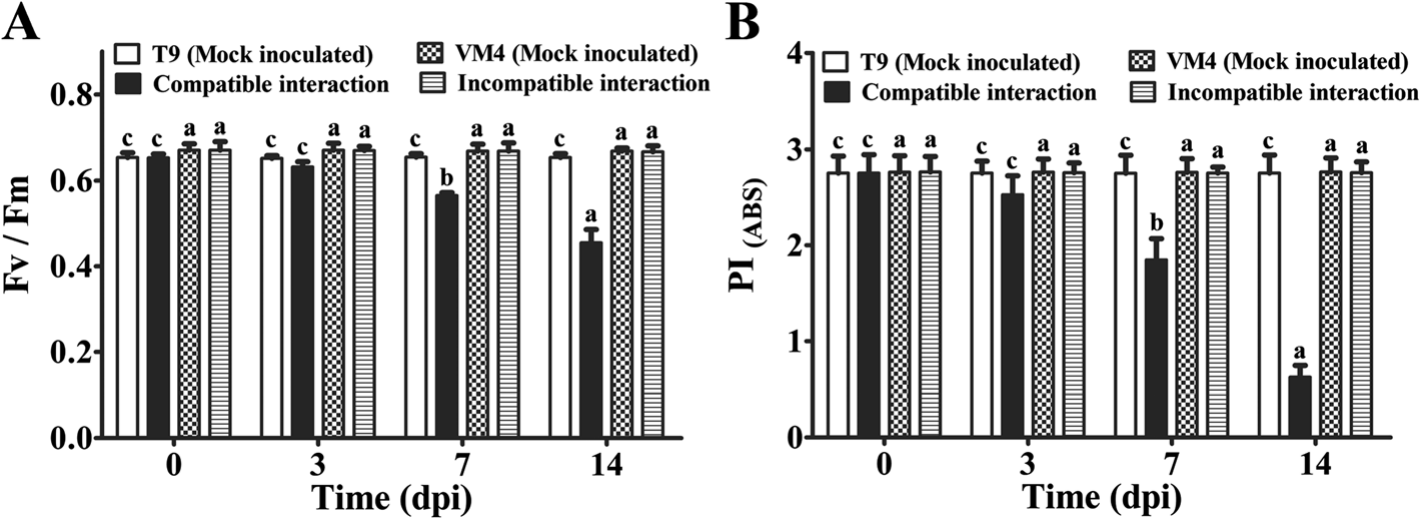
**Photosynthetic performance at different time point in compatible and incompatible MYMIV interaction.** Optimal quantum yield (Fv/Fm) (**A**). Performance Index (PI_ABS_)_._ (**B**) Bars indicate mean ± SD. The ANOVA and DMRT were performed between mock inoculated T9 and compatible interaction; and mock inoculated VM4 vs. incompatible interaction. Bars followed by same alphabets are not significantly different at p ≤ 0.05.

According to the phenomenological flux models, absorbance maxima per excited cross-section (ABS/CS_m_) declined upon virus infection during compatible interaction thereby lowering the trapped energy per cross-section (TR_0_/CS_m_) and electron transported per cross-section (ET_0_/CS_m_) (Additional file 3: Figure S3A). Significant changes in these parameters were not observed during incompatible interaction (Additional file 3: Figure S3B). MYMIV infection significantly decreased the ratio of variable and maximum fluorescence (F_v_/F_m_) during compatible interaction at 7 and 14 dpi (Figure 3A). The Performance Index at equal absorption (PI_ABS_) gradually declined in the susceptible genotype upon virus infection during establishment of disease (Figure 3B). All these parameters were comparable in two mock inoculated genotypes.

### Kinetics of antioxidant enzymes

**Figure 4:**
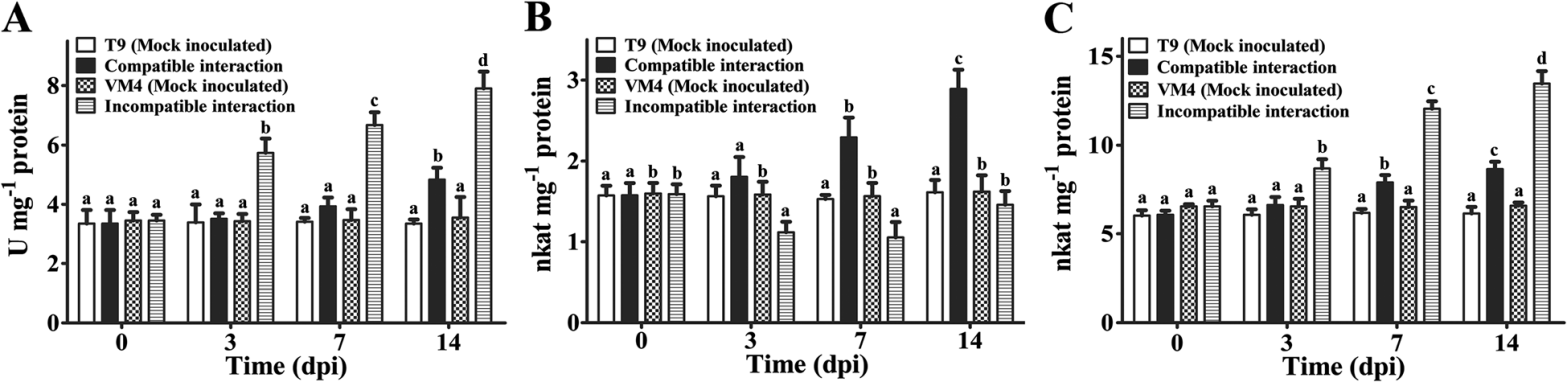
**Effect of MYMIV inoculation on antioxidant enzyme activity (SOD, POD and APX) ofV. mungo leaves.** (**A**) CAT (**B**) and APX (**C**) activity of *V*. *mungo* leaves. Bars indicate mean ± SD. The ANOVA and DMRT were performed between mock inoculated T9 and compatible interaction; and mock inoculated VM4 vs. incompatible interaction. Bars followed by same alphabets are not significantly different at p ≤ 0.05.

The activity of SOD sharply increased upon MYMIV infection at 3 dpi during incompatible interaction and gradually increased upto 2.3-fold at 14 dpi compared to that of the control. While there was a steady increase in the SOD activity during compatible interaction from 7 dpi onwards and significant level was attained at 14 dpi (Figure 4A).

CAT activity declined significantly at 3 and 7, dpi as compared to that of the control during incompatible interaction (Figure 4B). Whereas CAT activity increased significantly from 7 dpi during compatible interaction and 1.8-fold increment was noted at 14 dpi.

The activity of APX was induced significantly from 3 dpi during incompatible interaction, whereas significant increase in APX activity was recorded from 7 dpi onwards during compatible interaction (Figure 4C). There were negligible changes in SOD, CAT and APX activities in two mock inoculated genotypes at three time points (Figure 4A-C).

### Changes in the leaf proteome of *V. mungo* following MYMIV-infection

**Figure 5:**
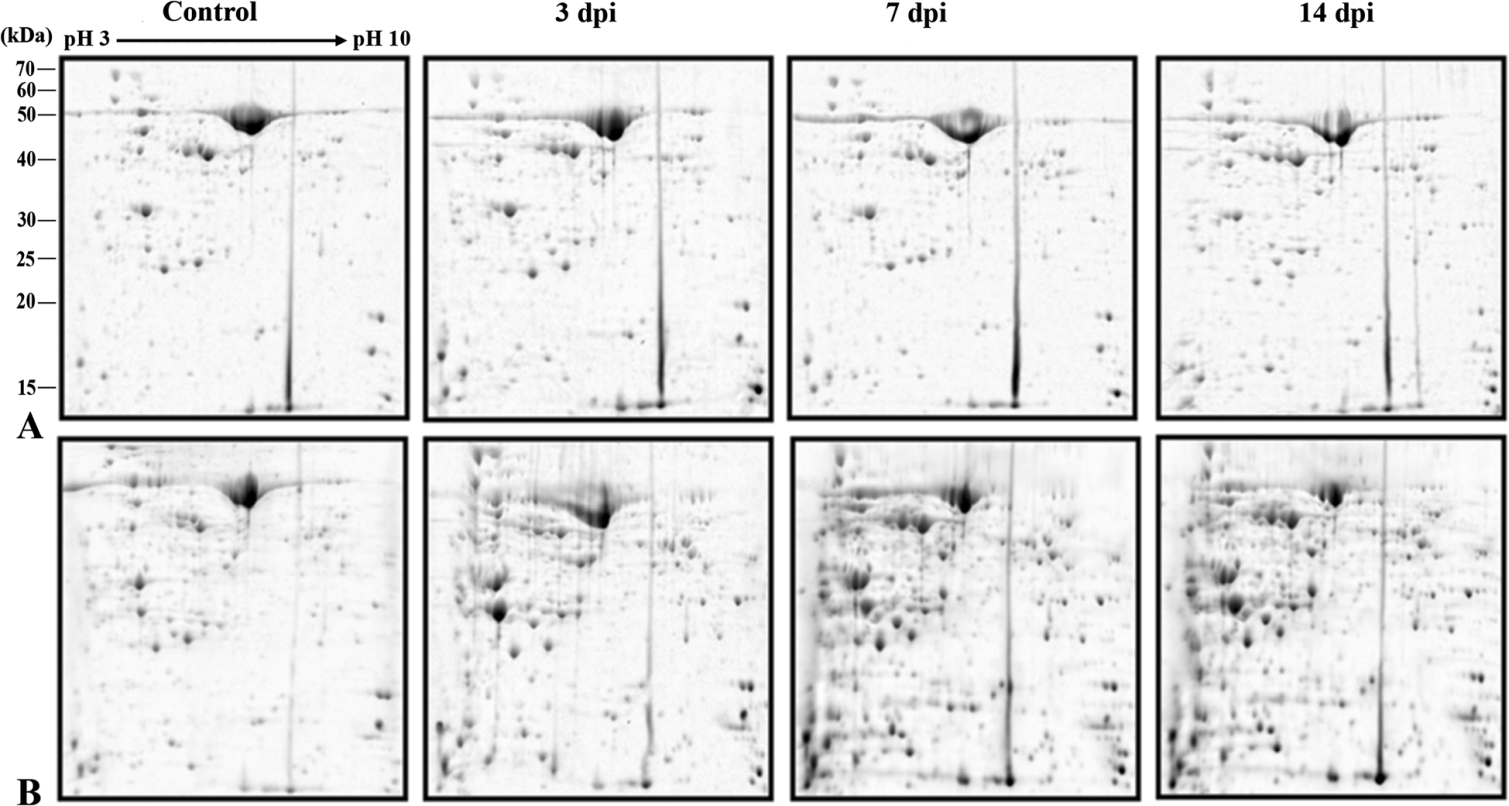
**Representative 2 DE gels of control, compatible and incompatible interaction between MYMIV and V. mungo leaf proteome.** Compatible interaction (**A**); Incompatible interaction (**B**).

A comprehensive leaf proteome analyses of MYMIV-susceptible and -resistant genotypes was done at 3, 7 and 14 dpi to elucidate qualitative and quantitative changes in the plant proteomes during compatible and incompatible interactions following MYMIV-infection. The representative 2-DE gels of control and virus inoculated protein samples from the susceptible and resistant genotypes are shown in Figure 5. About 350 protein spots were detected by analyses of the gels through normalization using local regression model. Among these, 150 spots were with reproducible differences in abundance (between and outside 2-fold limits) in all replicate gels between the virus-inoculated and control samples from susceptible (T9) and -resistant (VM4) genotypes. 109 such proteins were identified by MALDI-MS and MS/MS (PRIDE database Accession no. 15318). The average spot intensity, coefficient of variation (CV) and fold change of all the identified spots during compatible and incompatible interactions are represented in Additional file 4: Table S1. The CV < 43% was observed for all the spots at different time points between the same groups. Among these, CV < 30% was obtained for 74% of the identified spots. This range is consistent with the previous reports of average CV for biological replicate gels [9]. The identified proteins were functionally grouped according to Universal Protein Resource (Uniprot) database and in consultation with all available literatures (Additional file 5: Figure S4; Additional file 6: Table S2).

**Figure 6:**
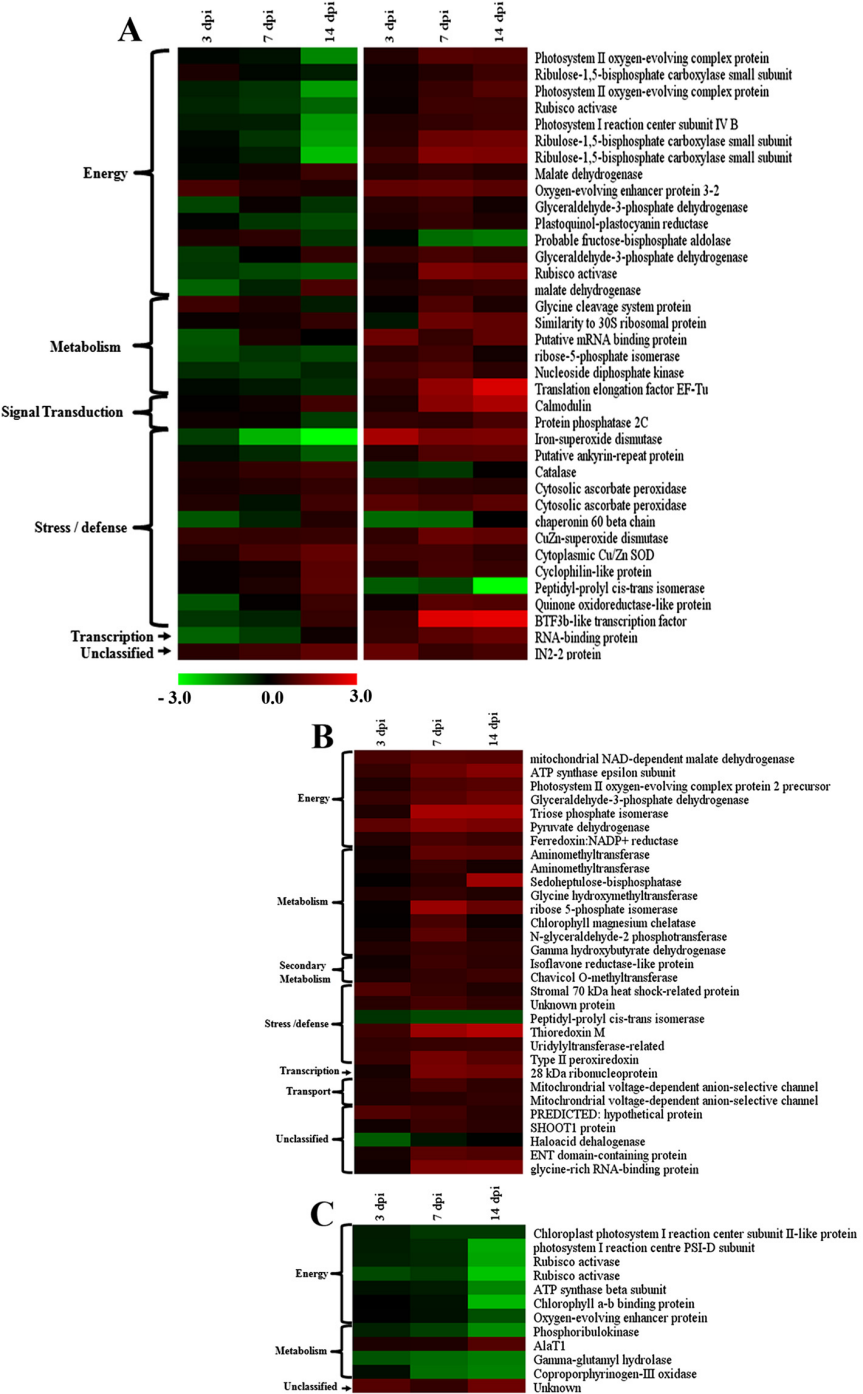
**Heat map profile of differentially abundant proteins on compatible and incompatible interaction between MYMIV and *V. mungo *leaf proteome.** Between compatible and incompatible interactions (**A**); only during incompatible interaction (**B**); only during compatible interaction (**C**). Protein abundance ratios (log 2 base transformed) are color coded with red indicating increased in abundance by MYMIV infection; green indicating decrease in abundance by the virus infection; and black indicating no change. Protein lists were clustered according to their functional category.

A specific criterion was applied to the identified proteins for the selection of differentially abundant spots. A protein was selected as differentially regulated only when its level differed at least ±1.5-fold (i.e., ratio ≥ 1.5 or ≤ 0.67) in all the replicate biological samples at one time point in two comparable sets. The distribution of differentially abundant proteins during compatible- and incompatible interactions are summarized in Additional file 7: Figure S5 that depicts an overlap of 37 proteins which are differentially regulated irrespective of types of plant-pathogen interaction. Differential proteins with significant fold change values after log conversion were analyzed by Multi-Experiment Viewer (MEV) software 4.8.1 and heat map profiles were generated (Figure 6). Levels of 29 proteins were upregulated significantly during incompatible interaction, except for peptidyl-prolyl cis-trans isomerase and haloacid dehalogenase (Figure 6B). During compatible interaction, most of these proteins were down-regulated, except AlaT1 and an unknown protein (Figure 6C).

**Figure 7:**
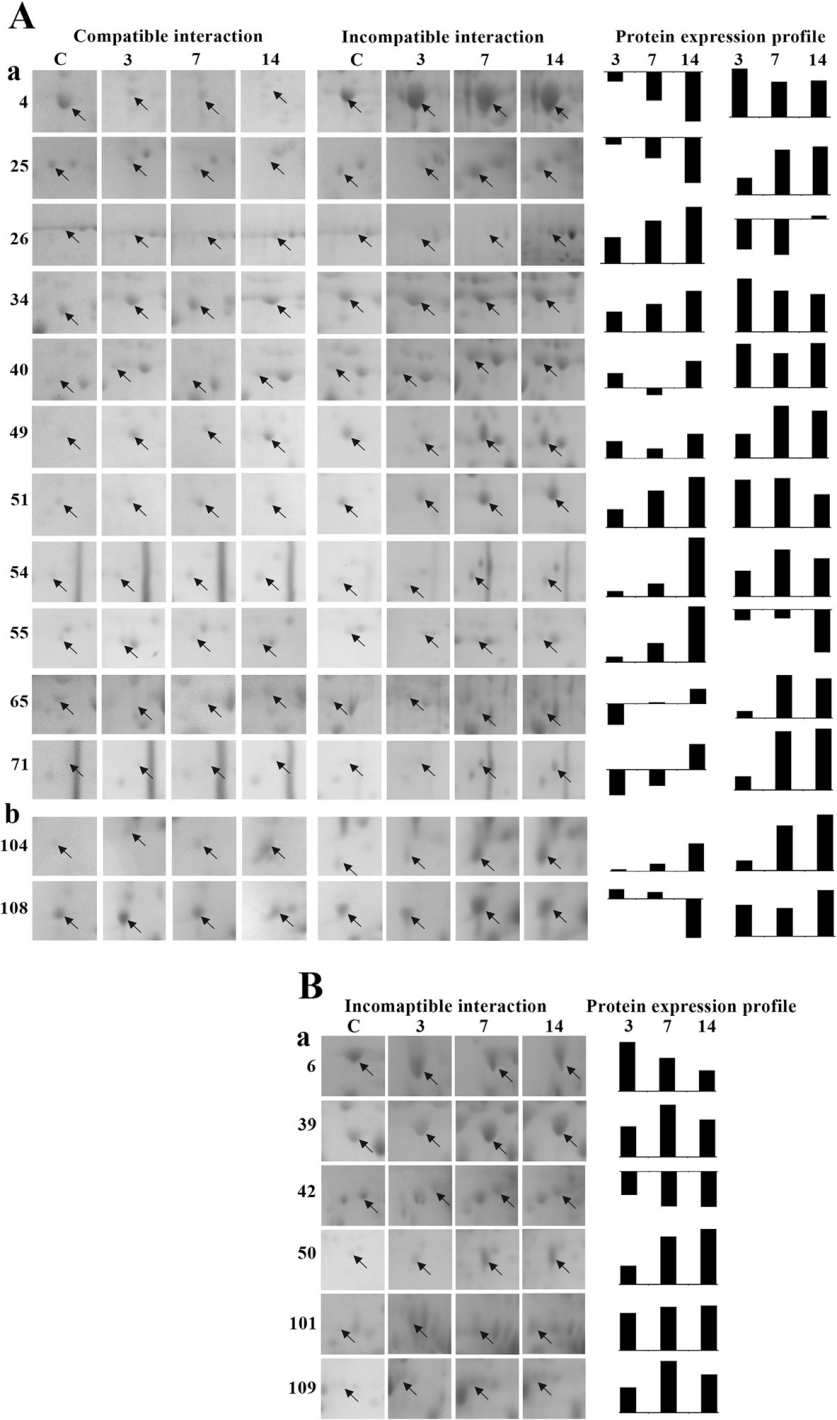
**Differentially abundant proteins on 2 DE gels during compatible and incompatible interaction between MYMIV and *V. mungo*leaf proteome.** (**A**); only during incompatible interaction (**B**). Stress/defense related proteins (**a**); Signal transduction related proteins (**b**). Arrows indicate proteins whose expression changed in response to virus infection.

To elucidate the plant-virus interactions we have concentrated mainly on the downstream analysis of the stress/defense related and signal transduction related proteins that play a crucial role in alleviating disease reactions of inoculated plants. The changes in the regulations of these proteins leading to differential abundance during both compatible and incompatible interactions were observed (Figure 7).

### Robust induction of stress/defense and signal transduction related proteins

**Figure 8:**
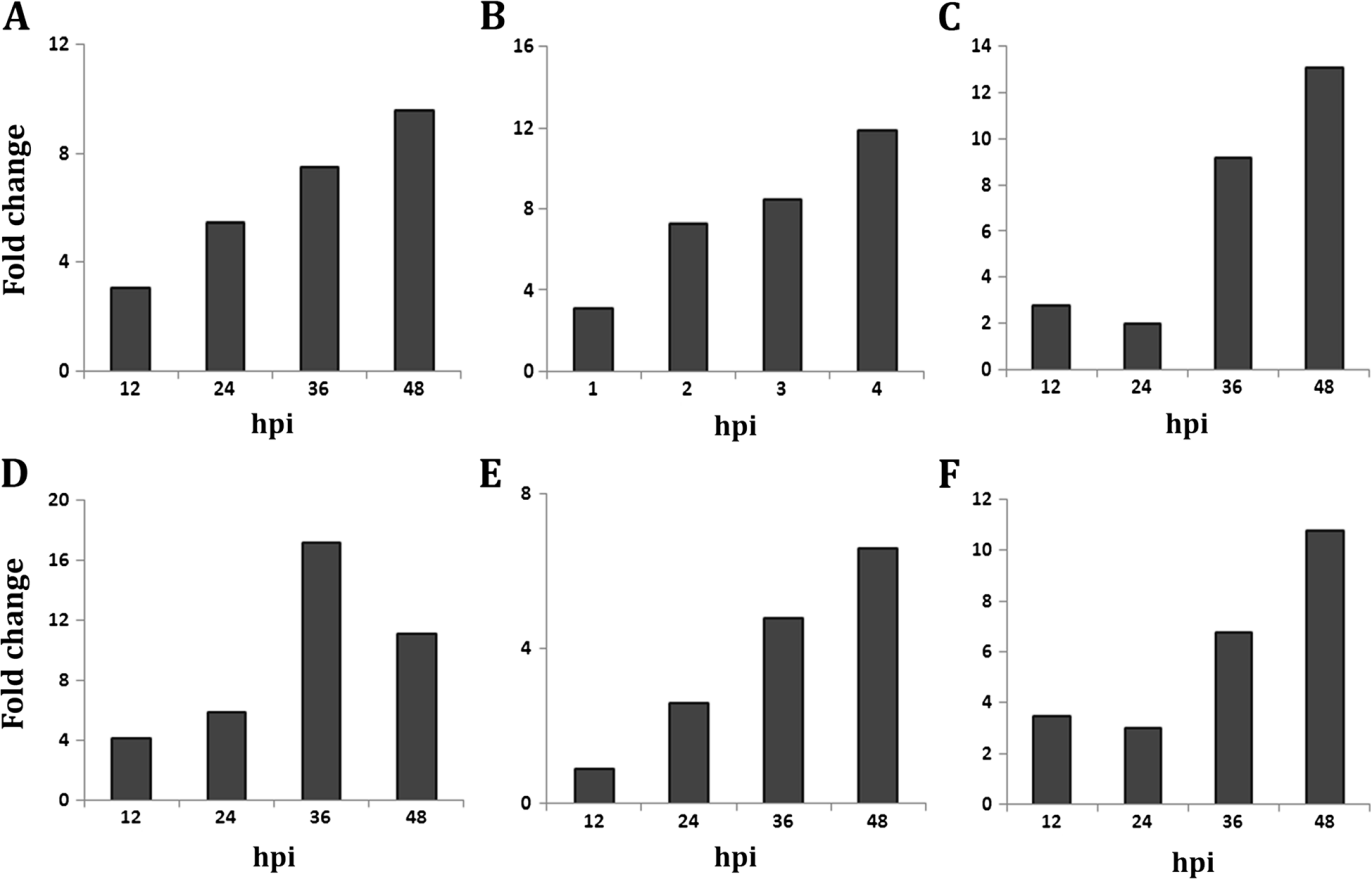
**Expression analysis of MYMIV responsive genes during MYMIV - *V. mungo *incompatible interaction by qRT-PCR.** (**A**) Superoxide dismutase (HO223986), (**B**) Thioredoxin (JG016041), (**C**) Ascorbate peroxidase (HO224008), (**D**) Calmodulin (JK086513), (**E**) Rubisco activase (HO223989) and (**F**) Pathogenesis related protein 1 (HO223973).

The production of ROS during plant pathogen interaction is harmful for the pathogen; ROS transmits signal to induce defense pathways in the host [10]. Among different isoforms of SOD, the level of Fe-SOD (spot no. 4) was highest (log 2 value, 1.97) at 3 dpi during incompatible interaction. The Fe-SOD decreased in abundance during compatible interaction. While, other two isoforms Cu/Zn SOD (spot nos. 49 and 51) increased in abundance during both compatible and incompatible interactions; but the magnitude of upregulation was higher in the resistant genotype. Data on SOD activities of *V. mungo* generated through biochemical investigation during compatible and incompatible interactions also corroborate with this proteomic information. The level of CAT (spot no. 26) was decreased in the resistant genotype at 3 and 7 dpi, whereas it increased in the susceptible genotype at all time points as also found in enzyme assay. Evidently, by up regulation of the activities as well as abundance of both the isoforms of SOD with simultaneous inhibition of CAT, a rapid transient up surge of H_2_O_2_ is expected in the resistant genotype. Such up surge in H_2_O_2_ activity was also found at 3 dpi in biochemical investigation. Presumably such transitions trigger signaling pathway to induce cascade of defense related gene expression. The uncontrolled production of ROS may be detrimental for the host primarily because it can also cause damage to its own cellular components [10]. Therefore, a balance needs to be maintained in order to protect host cells from the oxidative damage. Adequate upregulation of the ESTs homologous to antioxidants including SOD (GenBank ID. HO223986; Figure 8A), thioredoxin (GenBank ID. JG016041; Figure 8B) in the resistant genotype provide substantial evidence towards maintenance of ROS homeostasis within the resistant host. The proteomic and enzymatic analyses clearly discriminate the relative changes in APX (spot no. 34 and 40) level in resistant and susceptible genotypes. APX protein level was higher in resistant genotype upon virus inoculation, compared to the susceptible one, suggesting a better regulation of ROS metabolism during incompatible interaction. Simultaneously it was also revealed that transcripts of APX (GenBank ID HO224008; Figure 8C) increased drastically from 36 hrs during incompatible interaction.

Type II peroxiredoxin (spot no. 109) also increased in abundance at different time points in the resistant genotype upon virus inoculation. It is involved in ameliorating pathogen-induced oxidative stress and offers higher tolerance against pathogens [11]. Significant change in the level of peroxiredoxin was not found during compatible interaction.

Levels of uridylyltransferase-related protein (spot no. 101), quinine oxidoreductase-like protein (spot no. 65) and BTF3b-like transcription factor (spot no. 71) were enhanced during incompatible interaction, which are usually associated with abiotic stress tolerance. But in this case, presumably to combat with drought condition due to rapid efflux of anions led to depolarization of plasma membrane potential which regulates K^+^ ion channels resulting in extrusion of K^+^ ions. Thus, water potential decreased within the cell and created drought like situation upregulating drought responsive proteins.

The BTF3b-like transcription factor increased 6.8-fold (log 2 value, 2.76) at 14 dpi. These findings support the opinion that mechanisms by which plants respond to abiotic and biotic stresses are not independent, rather are involved in a common network through a continuous process of cross-talk [12,13].

The level of Calmodulin (CaM) (spot no. 104) was much higher during incompatible interaction as compared to compatible interaction in *V. mungo.* This is also supported by CaM (GenBank ID. JK086513) transcript analysis in resistant genotype of *V. mungo*, where transcripts were highly overexpressed during incompatible interaction (Figure 8D). CaM is an acidic Ca^2+^-binding protein that is induced upon various stresses. In response to the biotic stress, the cytosolic Ca^2+^ concentration in plants elevates rapidly via an increased Ca^2+^ influx, and then quickly returns to the basal level by Ca^2+^ efflux thus producing a Ca^2+^ spike [14]. The activated Ca^2+^-CaM complex binds to several target proteins and modulates different cellular processes [15]. Burst in cytosolic Ca^2+^ fluxes triggers induction of defense responses probably via calmodulin dependent kinases (CAM kinases) or calcium dependent protein kinases (CDPKs), the involvement of CDPKs in salicylic acid mediated defense response in *V. mungo* against MYMIV has been shown by us previously [8]. From the above findings it is envisaged that the ROS homeostasis in resistant genotypes of *V. mungo* is controlled by Ca^2+^-dependent pathway as also predicted by Grant and Loake [16].

The increased level of protein phosphatase 2C (PP2C) (spot no. 108) during incompatible interaction may trigger signal transduction pathway and consequently induces resistance response against MYMIV in *V. mungo*. It has been reported earlier that over expression of PP2C gene in transgenic tobacco plants resulted in increased disease resistance against tobacco mosaic virus [17]. This protein may be responsible for resistance mediated defense which in addition to recognizing the effector molecules of pathogen also induces defense response mainly activating PR proteins in the resistant plants, for which the signaling pathway is still inadequately deciphered.

### Induction of SAR through ankyrin repeat protein, a possibility

In the present study, level of an ankyrin repeat like domain containing protein (AKR, spot no. 25) was up-regulated upon MYMIV infection during incompatible interaction; whereas, it was gradually down-regulated in the MYMIV-susceptible genotype with the progression of the disease. In *Arabidopsis*, *NPR1* gene encodes an oligomeric AKR, a cytosolic protein. It has been reported that upon pathogen infection NPR1 oligomer converts into monomeric form and is transported to the nucleus to orchestrate expression of *PR* genes [18]. The induction of PR proteins ultimately contributes to SAR [19]. During the development of SAR, two isoforms of thioredoxin (Trx) regulate the conformation of NPR1 in the cellular redox environment [20]. Level of Trx M (spot no. 50) was also enhanced during incompatible interaction while, there was no significant change in the level during compatible interaction, suggesting both of these proteins are implicated in conferring MYMIV-resistance in *V. mungo*. *V. mungo* EST (GenBank ID. JG016041) homologous to *V. unguiculata* Trx, over-expressed during incompatible interaction with MYMIV (Figure 8B).

### MYMIV-stress predominately affects energy-related proteins

In the susceptible plants, establishment of YMD and appearance of the characteristic symptoms in the form of yellow mosaic should normally correspond with simultaneous decrease in the levels of proteins that are involved in photosynthesis and photorespiration. Evidently, chlorophyll fluorescence induction kinetics of MYMIV infected *V. mungo* plants indicates decrease in photosynthesis efficiency with gradual increase of chlorotic patches (Figure 3A, B). The photosynthetic machinery of susceptible plant is a primary target of MYMIV which is evident from the reduction in the photosynthetic rate and chlorophyll content upon virus infection (Additional file 2: Figures S2; Additional file 3: Figures S3). High turnover of photosynthetic proteins such as rubisco activase (spot nos. 5 and 73), Photosystem II oxygen-evolving complex protein (spot nos. 1, 3 and 56) and Ribulose-1, 5-bisphosphate carboxylase small subunit (spot no. 18 and 20) during incompatible interaction suggests that resistant plants evoke defense mechanisms to protect the photosynthetic machinery and enhance photosynthetic efficiency upon virus infection. Transcript analysis of rubisco activase (GenBank ID. HO223989) also supports this data (Figure 8E). Such increase in photosynthetic efficiency of MYMIV-resistant plants generates extra energy to avoid pathogen effector molecules. Treatment of SA also increases the photosynthetic efficiency of susceptible plants simultaneously increasing tolerance to MYMIV as demonstrated earlier [8].

The pyruvate dehydrogenase and malate dehydrogenase are important enzymes of tricarboxylic acid (TCA) cycle (Additional file 8: Figure S6). During incompatible interaction, pyruvate dehydrogenase (spot no. 77) was increased in abundance, but in the susceptible genotype it did not changed significantly. Levels of all three isoforms of malate dehydrogenase (spot nos. 24, 38, and 84) were increased in resistant genotype in contrast to the down-regulation in susceptible genotype. In the light of the earlier reports it is presumed that the up-regulation of these proteins triggers the TCA cycle to provide additional energy for defense response through the generation of pyruvate and NADPH [21].

### Redirecting carbohydrate flux towards pentose phosphate pathway

Researchers have shown that the resistance to pathogens involves activation of multiple metabolic pathways to support cellular energy requirements [22,23]. In the present study, several proteins involved in primary metabolism underwent significant changes in expression levels upon MYMIV infection. Among these, four enzymes of the pentose phosphate pathway (PPP), including ribose-5-phosphate isomerase (spot nos. 48 and 89), gamma hydroxybutyrate dehydrogenase (spot no. 86), a hypothetical protein (spot no. 47) and sedoheptulose-bisphosphatase (spot no. 43) were increased exclusively during incompatible interaction (Additional file 9: Figure S7). Such differential regulation suggests a redirection of metabolic flux towards PPP, possibly an adaptive strategy to survive under oxidative stress [24]. Thus, our conjecture is that the induction of the PPP is an active response of resistant genotype against MYMIV infection to enhance resistance. This proposition is also supported by the increased level of soluble sugar during incompatible interaction (Figure 1A). The enhancement of defence reactions is most likely mediated by the dual role of soluble sugars being fuel for metabolism and signal for defense-related genes activation.

### Secondary metabolism related proteins

The chavicol-o-methyltransferase (spot no. 63), a protein involved in phenylpropanoid pathway was increased significantly upon virus infection in resistant genotype. Level of this protein was declined in susceptible genotype. Induction of phenylpropanoid pathway also leads to the accumulation of salicylic acid, phytoalexins, antimicrobials, proline rich cell wall precursor, glycoproteins etc. all of which orchestrate together in the defense response against the pathogen.

Isoflavone reductase-like protein (spot no. 17), one of the key enzymes of isoflavonoid biosynthesis was up-regulated only during incompatible interaction. The induction of secondary metabolism is also supported by increased level of total phenolic content during incompatible interaction (Figure 2B). This is an explicit evidence of the adaptive strategy of the resistant plant against MYMIV stress.

### Unclassified proteins

Among the unclasssified proteins, level of a glycine-rich protein, GRP (spot no. 95) was significantly increased in the resistant genotype, but not in the susceptible plants upon MYMIV infection. GRP adheres tightly with the cell wall polysaccharides and is reported to be involved in cell wall repair [25]. Level of an ENT (EMSY N-terminal) domain containing protein (spot no. 93) increased significantly only during incompatible interaction, which has been implicated in chromatin remodeling [26]. In *Arabidopsis* EMSY-like proteins were reported to be involved in race specific immunity [27]. Further work on this protein may reveal some novel information regarding plant pathogen interaction.

### Biological network analysis of the differentially abundant proteins

**Figure 9:**
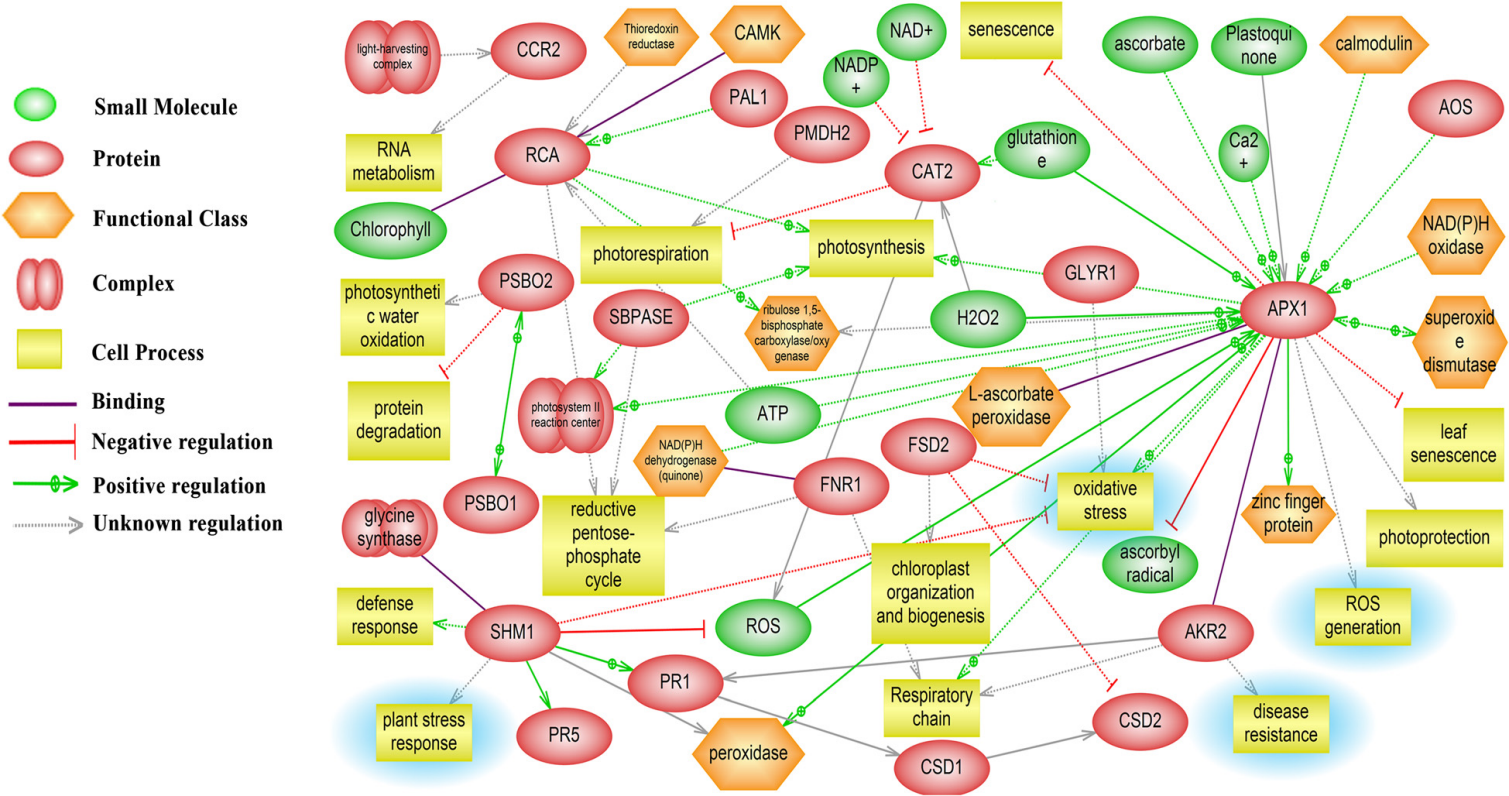
**Biological network of differentially abundant protein homologues constructed using the Pathway Studio software (version 7.1).** Proposed interactions are indicated by dashed lines (-----) and evidence-based interactions are indicated with solid lines (—). Abbreviations of the identified nodal proteins are CCR2: glycine rich RNA binding protein; RCA: rubisco activase; PSBO1/2: photosystem II oxygen evolving enhancer protein; SHM1: serine/glycine hydroxymethyltransferase; FNR1: ferredoxin: NADP(H) oxidoreductase; SBPASE: sedoheptulose-1,7-bisphosphatase; AKR2: ankyrin repeat protein; CSD1: copper/zinc superoxide dismutase; FSD2: Fe-superoxide dismutase; GLYR1: gamma-hydroxybutyrate dehydrogenase, APX1: ascorbate peroxidase.

The Pathway Studio software was employed to establish the molecular interaction and regulation network based on high-throughput interaction datasets (ResNet) from the model organism *Arabidopsis*. The network of various cellular pathways that are involved in inducing defense response contains several conglomerated cores of nodal proteins, like hubs of a wheel (Figure 9). APX, AKR, rubisco activase and serine/glycine hydroxymethyl transferase are hubs with major connectivity. These nodal proteins play the role of key regulators in bringing about a coordinated defense response in a highly orchestrated manner. A noteworthy feature is that the hubs control interrelationships between several cellular processes including defense response, protein degradation, leaf senescence, reductive pentose phosphate pathway and photorespiration. In this network, several proposed functional conglomerations are combined with known interactions thereby indicating novel roles of previously reported proteins. For example, APX is primarily known to function in ROS management has been shown to inhibit leaf senescence as well. It can also directly interact with ankyrin repeat domain protein to induce PR proteins. On the contrary, serine/glycine hydroxymethyl transferase, the positive regulator of PR proteins (PR1 and PR5) inhibits ROS generation suggesting a complex interaction in ROS regulation to maintain cellular redox homeostasis. Transcript analysis data has also shown an increase in PR1 transcripts during incompatible interactions (Figure 8F). The rubisco activase protein besides it known role in regulation of photosynthesis can also regulate the reductive PPP. Increase in rubisco activase both in transcript and proteome level also supports up-regulation of PPP.

## Conclusions

In this study, we have reported for the first time the protein profiling during compatible and incompatible interactions between MYMIV and *V*. *mungo*. Biochemical and proteomic analyses revealed early accumulation of the defense/stress related proteins involved in ROS metabolism during incompatible host-virus interaction triggers MYMIV-resistance. An increase in SOD activity with concurrent decrease in CAT activity results in increased accumulation of H_2_O_2_ which restrict virus multiplication in the resistant genotype. The consequent oxidative stress to the plant is countered by increased activity of APX. In the susceptible genotype, a decrease in the amount of proteins involved in the energy metabolism was observed after MYMIV inoculation attributed to the appearance of disease symptoms. Presumably ankyrin repeat domain containing protein serves as a nodal protein inducing SAR activity by interacting with several disease resistant proteins as shown in the biological network. Remarkably, during defense response redirection of carbohydrate flux towards pentose phosphate pathway occurred in resistant genotype of *V. mungo*. High turnover of photosynthetic proteins during incompatible interaction suggests that resistant plants evoke defense mechanisms to protect the photosynthetic machinery and enhance photosynthetic performance upon virus infection. Revelation of differential abundance of key cellular and regulatory proteins upon MYMIV stress elucidates molecular mechanism of defense response in *V. mungo* and establishment of the YMD. The data presented here is in concurrence with the view that partial over-lapping of different stress responses takes place and several cross-talk points are revealed.

## Methods

### Plant materials and virus inoculation

Seeds of *V*. *mungo* Cv. T9 (MYMIV-susceptible) obtained from the Pulse and Oilseed Research Station, Behrampur, Murshidabad, India and MYMIV-resistant genotype, VM4 were used in this study [7,28]. Whiteflies (*Bemisia tabaci*), the vector of MYMIV, were reared at the insectry room, Madhyamgram Experimental Farm, Bose Institute, Kolkata and virus inoculation was performed as described previously [8]. Three-week-old plants (T9 and VM4) were inoculated either with non viruliferous whiteflies (mock inoculation) or viruliferous whiteflies. The leaf tissues of both mock and MYMIV inoculated samples were harvested at 0, 3, 7 and 14 days post inoculation (dpi) for subsequent analyses.

### Determination of chlorophyll and carotenoid content

Chlorophyll a (Chl a), chlorophyll b (Chl b) and total carotenoids were determined spectrophotometrically [29]. The leaves extracted with 85% (v/v) aqueous acetone were centrifuged at 4000 rpm for 10 min. After suitable dilution, the extinction coefficient of the supernatant was measured at three wavelengths of 452.5, 644 and 663 nm using a Beckman Coulter DU® 520 UV–vis spectrophotometer (USA).

### Estimation of total carbohydrate content

Carbohydrate content was determined in the aqueous solution with anthrone sulfuric acid reagent [30]. The amount of insoluble carbohydrates was calculated from the difference between the amount of the total and soluble carbohydrates.

### Hydrogen peroxide and total phenolic content

Hydrogen peroxide content was measured according to Velikova et al. [31]. Total phenolic content was measured following Folin–Ciocalteau method [32].

### Antioxidant enzyme assays

Leaf tissues (0.5 g) were ground in a pre-chilled mortar and pestle in liquid nitrogen with 5 mL extraction buffer (50 mM potassium phosphate buffer pH 7.5, 1 mM EDTA, 1 mM PMSF). The homogenate was centrifuged at 8000 rpm for 15 min at 4°C and the supernatant collected. The activity of SOD was assayed by measuring the inhibition of photoreduction of nitro-blue tetrazolium [33]. One unit of enzyme activity is defined as the amount of enzyme required to inhibit the NBT reduction by 50%. The activity of CAT was determined spectrophotometrically by measuring the rate of H_2_O_2_ disappearance at 240 nm, taking Δε at 240 nm as 39.4 mM^−1^ cm^−1^[34]. The APX was assayed as described by Nakano and Asada [35]. APX and CAT activity was expressed as nkat mg^−1^ protein.

### Measurement of fluorescence induction kinetics

The Chlorophyll fluorescence induction kinetics was measured using a Plant Efficiency Analyzer (PEA, Hansatech, King's Lynn, Northfolk, England) [36]. The leaves were dark adapted for 30 min and the transient was induced by the red light of about 3400 μmol (photon) m^-2^s^-1^ provided by an array of 3 light-emitting diodes (peak 650 nm). The fluorescence signal is received by the sensor head during recording and is digitized in the control unit using a fast digital converter. The variable fluorescence (F_v_ = F_m_ – F_0_) and the ratio of variable and maximum fluorescence (F_v_/F_m_) were calculated. The energy pipeline model was prepared using the Biolyzer HP 3 software (the chlorophyll fluorescence analyzing program by Bioenergetics Laboratory, University of Geneva, Switzerland).

### Leaf proteome isolation and 2-DE

The leaf proteome was isolated according to Isaacson et al. with minor modifications [8,37]. The detailed experiments are described in the MIAPE (http://miapegeldb.expasy.org/ experiment/129/). Tissue of the samples collected from five independent plants were pooled to isolate protein and considered as one biological replicate to minimize plant to plant variations. Three biological replicates were also prepared for control and each of the infected samples. Protein concentration was estimated following the method of Bradford [38]. The 2-DE was performed according to the manufacturer’s recommendation (Bio-Rad). After electrophoresis, the gels were stained by Coomassie Brilliant Blue stain (0.1% Coomassie Brilliant Blue G-250, Sigma, in 50% v/v methanol/ 10% v/v acetic acid) for 12 h on an orbital shaker. Thereafter, the gels were rinsed with distilled water, followed by agitation in a destaining solution. The gels were stored in 5% acetic acid at 4°C for further analysis.

### Image acquisition and statistical analysis

Coomassie stained 2-D gels were digitalized using VersaDoc™ (Model 4000) Imaging System (Bio-Rad) and analyzed with PDQuest Advanced™ 2-D Analysis software (version 8.0.1, Bio-Rad). Spots were detected automatically by the Spot Detection Parameter Wizard using the Gaussian model with keeping the background sensitivity level at 4. To compare spot quantities across gels accurately, non-expression-related variations in spot intensity was compensated through normalization by local regression model. Three well-separated gels of control and infected (3, 7 and 14 dpi) samples of both T9 and VM4 were used to create “replicate groups” and quantitative analyses were performed between the control group and each corresponding infected group. The relative change in protein abundance were estimated between limit (±2-fold) and outside limits (> + 2-fold or < −2-fold) with respect to control. The normalized coefficient of variation (CV) within the different gels in the same group was calculated to examine variation in spot quantity.

### Protein identification

The relevant protein spots were excised using EXQuest Spot Cutter (Bio-Rad) from the 2-D gels. In-gel digestion was performed as previously described [39]. Mass spectra were obtained on an Autoflex II MALDI TOF/TOF (Bruker Daltonics, Germany) mass spectrometer equipped with a pulsed N_2_ laser (λ = 337 nm, 50 Hz), and database search performed as previously described [8].

### Functional annotation

The identified proteins were functionally annotated using Blast2GO V.2.4.3 software [40]. Protein sequences acquired from accession numbers assigned in mass spectrometric database were searched against NCBI non-redundant (NCBInr) database to perform BLASTp. The annotations of the identified proteins were made with default parameters. The assignment of identified the proteins into the different cellular pathways was done by the Kyoto Encyclopedia of Genes and Genomes (KEGG, http://www.genome.jp/kegg/kegg2.html) database in the Blast2GO software.

### Total RNA isolation and reverse transcription

Total RNA was extracted from MYMIV-inoculated leaves of resistant genotype at different (12, 24, 36 and 48) hours post inoculation using the RNeasy Plant Mini Kit (Qiagen, USA) following manufacturer’s instruction. Integrity of the RNA samples were checked in 1% agarose gel and quantified at 260 nm using the ND-1000 spectrophotometer (Nanodrop Technologies, USA). First strand cDNAs were synthesized from 2 μg total RNA using the revertAid first strand cDNA synthesis kit (Fermentas) following manufacturer’s protocol.

### Quantitative real-time RT-PCR (qRT-PCR) expression analysis

Time course expression profiles of six MYMIV-responsive transcripts were analyzed using qRT-PCR. Sequences of these ESTs were retrieved from the NCBI dbEST of a subtracted cDNA library of MYMIV- *V. mungo* incompatible interaction and primers were designed accordingly. The genes under consideration include Superoxide dismutase (SOD; HO223986), Thioredoxin (TRX; JG016041), Ascorbate peroxidase (APX; HO224008), Calmodulin (CaM; JK086513), Rubisco activase (RuAc; HO223989) and Pathogenesis related protein 1 (PR 1; HO223973). Two μl (50 ng/μl) of cDNA template was used in a 20 μl PCR mix containing 10 μl of SYBR Advantage qPCR Premix (2X) (Clontech) and 0.2 μm of custom designed forward and reverse primer. Real time PCR was carried out with iQ5 quantitative real time PCR system (Bio-Rad) under the following conditions: an initial denaturation at 95°C for 30 sec was followed by 40 cycles of 5 sec at 95°C, 30 sec at 60°C. On completion of each run, a dissociation curve analysis was done to check the specificity of the primers and absence of primer dimmers by heating the samples from 60 to 95°C. Raw fluorescence data were analysed by iQ5 Optical system software (Bio-Rad) and fold change was calculated using the 2^-ΔΔCt^ method [41] after normalization with the housekeeping gene actin.

### Protein network analysis

Pathway Studio 7.1 software (Ariadne Genomics, Rockville, MD, USA) was used to study functional interactions and possible pathways among the identified proteins [42]. The accession numbers (MSDB/Swissprot or NCBInr) of the significant proteins with differential abundance were converted to TAIR (The Arabidopsis Information Resource) IDs by performing TAIR BLAST 2.2.8. To establish the relationship between proteins and cellular processes, the TAIR IDs were imported to Pathway Studio 7.1 and an interaction network was constructed that included the upstream regulators and downstream targets of the proteins. Each identified cellular process was confirmed via the PubMed/Medline hyperlink embedded in each node.

## Competing interests

We hereby declare that we have no financial or non-financial competing interest.

## Authors’ contributions

AP designed and coordinated the study and edited the manuscript with in puts from SK. SK did almost all biochemical analyses, proteomics and bioinformatics work under the guidance of AP and initially drafted the manuscript. DC initiated and standardized the proteomics work also gave feedback drafting the manuscript. AK did RNA isolation, reverse transcription and expression analysis of genes by qRT-PCR and drafted the portion of his work. All authors participated in finalizing the text and approved the final manuscript.

## Authors’ information

SK did this work as part of his Ph.D. thesis and now working as a post-doc. DC did this work during his post-doc and now serving as an Assistant Professor in the Department of Bioscience and Biotechnology, Banasthali Vidyapith, Rajasthan 302044, India. AK has just submitted his Ph.D. thesis. AP is a Senior Professor in the Department of Plant Biology, Bose Institute, India and mainly involved in deciphering plant pathogen interactions at the genetical, genomic and proteomic level.

## Supplementary Material

Additional file 1 Symptoms development in leaves of susceptible genotypes and phenotype of resistant leaves following MYMIV inoculation. Click here for file

Additional file 2 **Effect of MYMIV inoculation on chl a, chl b and carotenoid content of *V. mungo *leaves.** Click here for file

Additional file 3 Energy pipeline leaf model of phenomenological fluxes (per cross-section) at different time points after MYMIV infection. Click here for file

Additional file 4 Comparative analysis of the identified protein spots during compatible and incompatible interactions. Click here for file

Additional file 5 Functional categorization of identified proteins according to Uniport database and available literature. Click here for file

Additional file 6 The leaf proteins identified by MALDI-MS and MS/MS analysis and categorized using Uniport database. Click here for file

Additional file 7 Venn diagrams depicting the distribution of differentially abundant proteins at different time points. Click here for file

Additional file 8 Involvement of the identified proteins in the tricarboxylic acid cycle pathway assigned by KEGG database in Blast2go software. Click here for file

Additional file 9 Involvement of the identified proteins in the pentose phosphate pathway assigned by KEGG database in Blast2go software. Click here for file

## References

[B1] Yang C Guo R Jie F Nettleton D Peng J Carr T Yeakley JM Fan JB Whitham SA Spatial analysis of *Arabidopsis thaliana* gene expression in response to Turnip mosaic virus infection Mol Plant Microbe In 2007 20 359 370 10.1094/MPMI-20-4-0358 17427806

[B2] Van Loon LC Pierpoint WS Boller T Conejero V Recommendations for naming plant pathogenesis–related proteins Plant Mol Biol Rep 1994 12 245 264 10.1007/BF02668748

[B3] Sels J Mathys J De Coninck BMA Cammue BPA De Bolle MFC Plant pathogenesis-related (PR) proteins: A focus on PR peptides Plant Physiol Biochem 2008 46 941 950 10.1016/j.plaphy.2008.06.011 18674922

[B4] Durrant WE Dong X Systemic acquired resistance Annu Rev Phytopathol 2004 42 185 209 10.1146/annurev.phyto.42.040803.140421 15283665

[B5] Oliveira JTA Andrade NC Martins-Miranda AS Soares AA Gondim DMF Araújo-Filho JH Freire-Filho FR Vasconcelos IM Differential expression of antioxidant enzymes and PR-proteins in compatible and incompatible interactions of cowpea (*Vigna unguiculata*) and the root-knot nematode *Meloidogyne incognita* Plant Physiol Biochem 2012 51 145 152 22153251 10.1016/j.plaphy.2011.10.008

[B6] Torres MA ROS in biotic interactions Physiol Plantarum 2010 138 414 429 10.1111/j.1399-3054.2009.01326.x 20002601

[B7] Basak J Kundagrami S Ghosh TK Pal A Development of yellow mosaic virus (YMV) resistance linked DNA marker in *Vigna mungo* from populations segregating for YMV–reaction Mol Breed 2005 14 375 383 10.1007/s11032-005-0238-6

[B8] Kundu S Chakraborty D Pal A Proteomic analysis of salicylic acid induced resistance to Mungbean Yellow Mosaic India Virus in *Vigna mungo* J Proteomics 2011 74 337 349 10.1016/j.jprot.2010.11.012 21130907

[B9] Molloy MP Brzezinski EE Hang J McDowell MT VanBogelen RA Overcoming technical variation and biological variation in quantitative proteomics Proteomics 2003 3 1912 1919 10.1002/pmic.200300534 14625853

[B10] Gara LD Pinto MC Tommasi F The antioxidant systems vis-à-vis reactive oxygen species during plant–pathogen interaction Plant Physiol Biochem 2003 41 863 870 10.1016/S0981-9428(03)00135-9

[B11] Kiba A Nishihara M Tsukatani N Nakatsuka T Kato Y Yamamura S A peroxiredoxin Q homolog from Gentians is involved in both resistance against fungal disease and oxidative stress Plant Cell Physiol 2005 46 1007 1015 10.1093/pcp/pci109 15840643

[B12] Valcu CM Junqueira M Shevchenko A Schlink K Comparative proteomic analysis of responses to pathogen infection and wounding in *Fagus sylvatica* J Proteome Res 2009 8 4077 4091 10.1021/pr900456c 19575529

[B13] Orsini F Cascone P DePascale S Barbieri G Corrado G Rao R Maggio A Systemin–dependent salinity tolerance in tomato: evidence for specific convergence of abiotic and biotic stress responses Physiol Plant 2010 138 10 21 10.1111/j.1399-3054.2009.01292.x 19843237

[B14] Evans NH McAinsh MR Hetherington AM Calcium oscillations in higher plants Curr Opin Plant Biol 2001 4 415 420 10.1016/S1369-5266(00)00194-1 11597499

[B15] Yang T Poovaiah BW Calcium/calmodulin–mediated signal network in plants Trends Plant Sci 2003 8 505 512 10.1016/j.tplants.2003.09.004 14557048

[B16] Grant JJ Loake GJ Role of reactive oxygen intermediates and cognate redox signaling in disease resistance Plant Physiol 2000 124 21 30 10.1104/pp.124.1.21 10982418 PMC1539275

[B17] Hu X Zhang H Li G Yang Y Zheng Z Song F Ectopic expression of a rice protein phosphatase 2C gene OsBIPP2C2 in tobacco improves disease resistance Plant Cell Rep 2009 28 985 995 10.1007/s00299-009-0701-7 19381642

[B18] Mou Z Fan W Dong X Inducers of plant systemic acquired resistance regulate NPR1 function through redox changes Cell 2003 113 935 944 10.1016/S0092-8674(03)00429-X 12837250

[B19] Dong X Genetic dissection of systemic acquired resistance Curr Opin Plant Biol 2001 4 309 314 10.1016/S1369-5266(00)00178-3 11418340

[B20] Tada Y Spoel SH Pajerowska–Mukhtar K Mou Z Song J Wang C Zuo J Dong X Plant immunity requires conformational changes of NPR1 via S–nitrosylation and thioredoxins Science 2008 32 952 956 18635760 10.1126/science.1156970 PMC3833675

[B21] Zulak KG Khan MF Alcantara J Schriemer DC Facchini PJ Plant defense responses in opium poppy cell cultures revealed by liquid chromatography–tandem mass spectrometry proteomics Mol Cell Proteomics 2009 8 86 98 10.1074/mcp.M800211-MCP200 18682378

[B22] Bolton MD Kolmer JA Xu WW Garvin DF Lr34–mediated leaf rust resistance in wheat: Transcript profiling reveals a high energetic demand supported by transient recruitment of multiple metabolic pathways Mol Plant Microbe In 2008 21 1515 1527 10.1094/MPMI-21-12-1515 18986248

[B23] Scheideler M Schlaich NL Fellenberg K Beissbarth T Hauser NC Vingron M Slusarenko AJ Hoheisel JD Monitoring the switch from housekeeping to pathogen defense metabolism in *Arabidopsis thaliana* using cDNA arrays J Biol Chem 2002 277 10555 10561 10.1074/jbc.M104863200 11748215

[B24] Grant CM Metabolic reconfiguration is a regulated response to oxidative stress J Biol 2008 7 1 10.1186/jbiol63 18226191 PMC2246036

[B25] Ringlia C Keller B Ryserb U Glycine–rich proteins as structural components of plant cell walls Cell Mol Life Sci 2001 58 1430 1441 10.1007/PL00000786 11693524 PMC11337278

[B26] Hughes-Davies L Huntsman D Ruas M Fuks F Bye J Chin SF Milner J Brown LA Hsu F Gilks B Nielsen T Schulzer M Chia S Ragaz J Cahn A Linger L Ozdag H Cattaneo E Jordanova ES Schuuring E Yu DS Venkitaraman A Ponder B Doherty A Aparicio S Bentley D Theillet C Ponting CP Caldas C Kouzarides T EMSY links the BRCA2 pathway to sporadic breast and ovarian cancer Cell 2003 115 523 535 10.1016/S0092-8674(03)00930-9 14651845

[B27] Tsuchiya T Eulgem T EMSY–like genes are required for full RPP7–mediated race–specific immunity and basal defense in *Arabidopsis* Mol Plant Microbe In 2011 24 1573 1581 10.1094/MPMI-05-11-0123 21830950

[B28] Kundagrami S Basak J Maiti S Kundu A Das B Ghosh TK Pal A Agronomic, genetic and molecular characterization of MYMIV tolerant mutant lines of *Vigna mungo* Int J Plant Breed Genet 2009 3 1 10 10.3923/ijpbg.2009.1.10

[B29] Metzner H Rau H Senger H Studies on synchronization of some pigment-deficient *Chlorella* mutants Planta 1965 65 1861 1894

[B30] Radwan DEM Fayez KF Mahmoud SY Hamad A Lu G Physiological and metabolic changes of *Cucurbita pepo* leaves in response to Zucchini yellow mosaic virus (ZYMV) infection and salicylic acid treatments Plant Physiol Biochem 2007 45 480 489 10.1016/j.plaphy.2007.03.002 17466528

[B31] Velikova V Yordanov L Edreva A Oxidative stress and some antioxidant systems in acid rain–treated bean plants: Protective role of exogenous polyamines Plant Sci 2000 151 59 66 10.1016/S0168-9452(99)00197-1

[B32] Singleton VL Orthofer R Lamuela–Raventos RM Analysis of total phenol and other oxidation substrates and antioxidants by means of Folin–Ciocalteu reagent Methods Enzymol 1999 299 152 178

[B33] Beauchamp C Fridovich I Superoxide dismutase: improved assays and an assay applicable to acrylamide gels Anal Biochem 1971 44 276 287 10.1016/0003-2697(71)90370-8 4943714

[B34] Miyagawa Y Tamoi M Shigeoka S Evaluation of the defense system in chloroplasts to photooxidative stress caused by paraquat using transformed tobacco plants expressing catalase from *Escherichia coli* Plant Cell Physiol 2000 41 311 320 10.1093/pcp/41.3.311 10805594

[B35] Nakano Y Asada K Hydrogen peroxide is scavenged by ascorbate- specific peroxidase in spinach chloroplasts Plant Cell Physiol 1981 22 867 880

[B36] Strasser RJ Srivastava A Govindjee Polyphasic chlorophyll a fluorescence transient in plants and cyanobacteria Photochem Photobiol 1995 61 32 42 10.1111/j.1751-1097.1995.tb09240.x

[B37] Isaacson T Damasceno CMB Saravanan RS He Y Catala C Saladie M Rose JKC Sample extraction techniques for enhanced proteomic analysis of plant tissues Nat Protoc 2006 1 769 774 10.1038/nprot.2006.102 17406306

[B38] Bradford MM A rapid and sensitive method for quantification of proteins utilizing the principle of protein dye binding Anal Biochem 1976 72 248 254 10.1016/0003-2697(76)90527-3 942051

[B39] Kundu S Chakraborty D Das KP Pal A An efficient in-gel digestion protocol for mass spectral analysis by MALDI-TOF-MS and MS/MS and its use for proteomic analysis of leaves of *Vigna mungo* Plant Mol Biol Rep 2013 31 47 54 10.1007/s11105-012-0475-x

[B40] Conesa A Gotz S Garcia–Gomez JM Terol M Talon M Robles M Blast2GO: a universal tool for annotation, visualization and analysis in functional genomics research Bioinformatics 2005 21 3674 3676 10.1093/bioinformatics/bti610 16081474

[B41] Livak KJ Schmittgen TD Analysis of relative gene expression data using real-time quantitative PCR and the 2^-ΔΔCt^ method Methods 2001 25 402 408 10.1006/meth.2001.1262 11846609

[B42] Nikitin A Egorov S Daraselia N Mazo I Pathway studio–the analysis and navigation of molecular networks Bioinformatics 2003 19 2155 2157 10.1093/bioinformatics/btg290 14594725

