# Supplementary material for: Proteomics approach combined with biochemical attributes to elucidate compatible and incompatible plant-virus interactions between Vigna mungo and Mungbean Yellow Mosaic India Virus
Source: Proteome Sci. 2013 Apr 15;11:15. doi: 10.1186/1477-5956-11-15 (PMC3639080; doi:10.1186/1477-5956-11-15)
Supplement: Additional file 1 — Symptoms development in leaves of susceptible genotypes and phenotype of resistant leaves following MYMIV inoculation. [file 1477-5956-11-15-S1.doc]

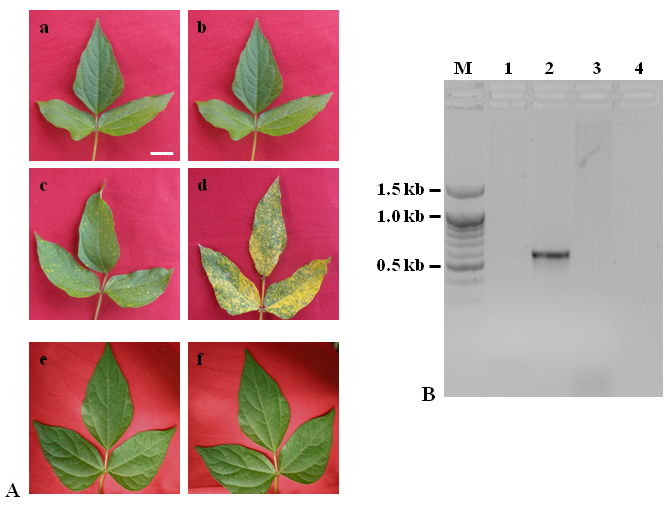
**Figure S1:** (A) The morphological appearance of susceptible (a-d) and resistant (e-f) genotypes leaves following MYMIV inoculation. a: Control; b: infected at 3 dpi; c: infected at 7 dpi; d: infected at 14 dpi; e: VM4 control; f: virus inoculated at 14 dpi (bar 1 cm). (B) 1.5 % agarose gel electrophoresis. Lane M: Molecular weight marker; Lane 1: Amplified DNA from T9 control (mock inoculated) leaf sample; Lane 2: Amplified DNA fragment of MYMIV-CP from infected sample (T9) at 14 dpi. Lane 3: Amplified DNA from VM4 control (mock inoculated) leaf sample; Lane 4: Amplified DNA from VM4 leaf sample at 14 dpi.
