# Supplementary material for: Proteomics approach combined with biochemical attributes to elucidate compatible and incompatible plant-virus interactions between Vigna mungo and Mungbean Yellow Mosaic India Virus
Source: Proteome Sci. 2013 Apr 15;11:15. doi: 10.1186/1477-5956-11-15 (PMC3639080; doi:10.1186/1477-5956-11-15)
Supplement: Additional file 2 — Effect of MYMIV inoculation on chl a, chl b and carotenoid content of V. mungo leaves. [file 1477-5956-11-15-S2.doc]

**
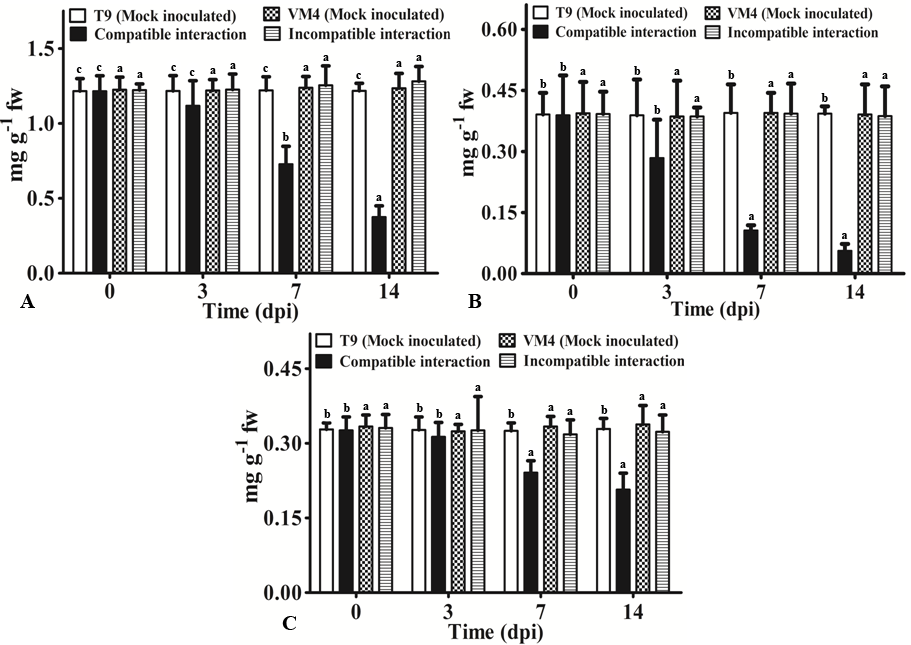
**

**Figure S2:** Effect of MYMIV inoculation on chl a (A) chl b (B) and carotenoid (C) content of *V*. *mungo* leaves. The ANOVA and DMRT were performed between mock inoculated T9 and compatible interaction; and mock inoculated VM4 vs. incompatible interaction. Bars followed by same alphabets are not significantly different at p≤0.05.
