# Supplementary material for: Proteomics approach combined with biochemical attributes to elucidate compatible and incompatible plant-virus interactions between Vigna mungo and Mungbean Yellow Mosaic India Virus
Source: Proteome Sci. 2013 Apr 15;11:15. doi: 10.1186/1477-5956-11-15 (PMC3639080; doi:10.1186/1477-5956-11-15)
Supplement: Additional file 3 — Energy pipeline leaf model of phenomenological fluxes (per cross-section) at different time points after MYMIV infection. [file 1477-5956-11-15-S3.doc]

**
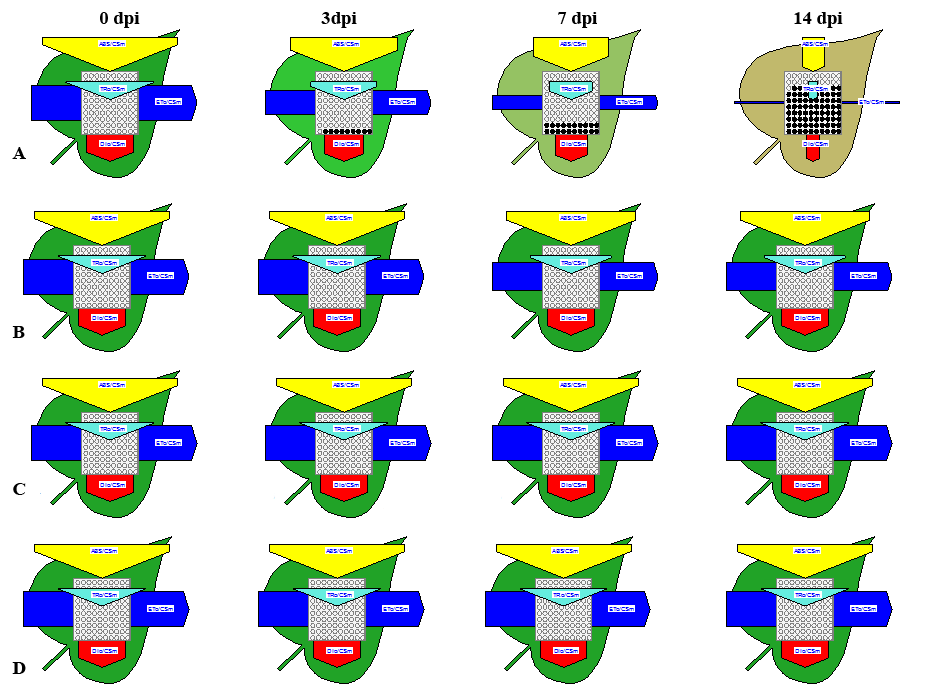
**

**Figure S3:** Energy pipeline leaf model of phenomenological fluxes (per cross-section) during compatible interaction (A) incompatible interaction (B) T9 mock inoculated (C) and VM4 mock inoculated (D) leaves at different time points. The value of each parameter has been represented in relative changes in width of each arrow. Active reaction centers (RC) are shown as open circles and inactive RCs are in closed circles.
