# Supplementary material for: Proteomics approach combined with biochemical attributes to elucidate compatible and incompatible plant-virus interactions between Vigna mungo and Mungbean Yellow Mosaic India Virus
Source: Proteome Sci. 2013 Apr 15;11:15. doi: 10.1186/1477-5956-11-15 (PMC3639080; doi:10.1186/1477-5956-11-15)
Supplement: Additional file 4 — Comparative analysis of the identified protein spots during compatible and incompatible interactions. [file 1477-5956-11-15-S4.doc]

**Table S1:** Comparative analysis of the identified spots during compatible and incompatible interactions.

| **Spot no** | **Control**  **(a)** | **N**  **(b)** | **CV**  **(c)** | **3 dpi**  **(a)** | **N**  **(b)** | **CV**  **(c)** | **Ratio**  **(d)** | **7 dpi**  **(a)** | **N**  **(b)** | **CV**  **(c)** | **Ratio**  **(d)** | **14 dpi**  **(a)** | **N**  **(b)** | **CV**  **(c)** | **Ratio**  **(d)** |
| --- | --- | --- | --- | --- | --- | --- | --- | --- | --- | --- | --- | --- | --- | --- | --- |
| **Compatible interaction** | | | | | | | | | | | | | | | |
| 1 | 127740.3 | 3 | 20.70% | 122764.3 | 3 | 22.70% | 0.96 | 111355.6 | 3 | 21.70% | 0.87 | 42529.5 | 3 | 10.50% | 0.33 |
| 2 | 255170.2 | 3 | 24.00% | 319792.6 | 3 | 22.00% | 1.25 | 241123 | 3 | 14.00% | 0.94 | 168721.2 | 3 | 14.30% | 0.66 |
| 3 | 426034.1 | 3 | 35.70% | 331171.8 | 3 | 25.70% | 0.78 | 285939.8 | 3 | 25.20% | 0.67 | 119117.7 | 3 | 15.40% | 0.28 |
| 4 | 133127.9 | 3 | 26.80% | 80885.5 | 3 | 27.80% | 0.61 | 30635.7 | 3 | 16.40% | 0.23 | 10635.7 | 3 | 21.30% | 0.08 |
| 5 | 441291.5 | 3 | 28.30% | 328363.6 | 3 | 26.30% | 0.74 | 286818.9 | 3 | 22.50% | 0.65 | 195130.8 | 3 | 38.30% | 0.44 |
| 6 | 51308 | 3 | 21.24% | 64024.5 | 3 | 22.24% | 1.25 | 75605.4 | 3 | 21.24% | 1.47 | 58610.5 | 3 | 22.00% | 1.14 |
| 7 | 47937.6 | 3 | 32.50% | 51402.1 | 3 | 22.50% | 1.07 | 67199.2 | 3 | 22.00% | 1.40 | 62561.3 | 3 | 12.20% | 1.31 |
| 8 | 95025.1 | 3 | 39.10% | 75964 | 3 | 35.10% | 0.80 | 60602.1 | 3 | 19.10% | 0.64 | 60602.1 | 3 | 29.10% | 0.64 |
| 9 | 128995.1 | 3 | 38.90% | 129035.2 | 3 | 34.90% | 1.00 | 124896.4 | 3 | 28.90% | 0.97 | 119703.4 | 3 | 32.90% | 0.93 |
| 10 | 263562.2 | 3 | 39.60% | 179310.2 | 3 | 36.60% | 0.68 | 198602 | 3 | 29.40% | 0.75 | 194095.4 | 3 | 32.60% | 0.74 |
| 11 | 211368.9 | 3 | 40.30% | 168721 | 3 | 30.50% | 0.80 | 150211.6 | 3 | 20.10% | 0.71 | 52775.1 | 3 | 20.30% | 0.25 |
| 12 | 228375.8 | 3 | 29.70% | 182515.7 | 3 | 29.60% | 0.80 | 179218.2 | 3 | 25.40% | 0.78 | 66818.3 | 3 | 19.70% | 0.29 |
| 13 | 281081.2 | 3 | 35.30% | 219481.4 | 3 | 25.60% | 0.78 | 203226.6 | 3 | 25.10% | 0.72 | 72521.8 | 3 | 32.30% | 0.26 |
| 14 | 220738.3 | 3 | 41.80% | 121097.2 | 3 | 31.80% | 0.55 | 140073.3 | 3 | 34.50% | 0.63 | 44635.7 | 3 | 35.80% | 0.20 |
| **Spot no** | **Control**  **(a)** | **N**  **(b)** | **CV**  **(c)** | **3 dpi**  **(a)** | **N**  **(b)** | **CV**  **(c)** | **Ratio**  **(d)** | **7 dpi**  **(a)** | **N**  **(b)** | **CV**  **(c)** | **Ratio**  **(d)** | **14 dpi**  **(a)** | **N**  **(b)** | **CV**  **(c)** | **Ratio**  **(d)** |
| 15 | 128995.1 | 3 | 37.40% | 99035.2 | 3 | 27.40% | 0.77 | 77896.4 | 3 | 27.20% | 0.60 | 41703.4 | 3 | 31.40% | 0.32 |
| 16 | 38694 | 3 | 27.60% | 28004.2 | 3 | 26.80% | 0.72 | 33888.5 | 3 | 37.40% | 0.88 | 50003.4 | 3 | 22.60% | 1.29 |
| 17 | 44521.7 | 3 | 24.40% | 31915.9 | 3 | 23.60% | 0.72 | 41158.8 | 3 | 24.40% | 0.92 | 53257.8 | 3 | 20.40% | 1.20 |
| 18 | 196275.7 | 3 | 33.60% | 182275.5 | 3 | 23.70% | 0.93 | 129417.5 | 3 | 23.30% | 0.66 | 52604.4 | 3 | 30.60% | 0.27 |
| 19 | 62926.9 | 3 | 18.50% | 58241.3 | 3 | 28.60% | 0.93 | 71271.7 | 3 | 13.20% | 1.13 | 80365.9 | 3 | 10.50% | 1.28 |
| 20 | 251727.4 | 3 | 32.80% | 243229.5 | 3 | 22.70% | 0.97 | 196419.2 | 3 | 22.80% | 0.78 | 53632 | 3 | 31.80% | 0.21 |
| 21 | 186275.7 | 3 | 27.70% | 162275.5 | 3 | 26.70% | 0.87 | 149417.5 | 3 | 24.20% | 0.80 | 62604.4 | 3 | 25.90% | 0.34 |
| 22 | 66577.9 | 3 | 40.90% | 92712.6 | 3 | 34.70% | 1.39 | 56461.4 | 3 | 34.90% | 0.85 | 79579.4 | 3 | 41.90% | 1.20 |
| 23 | 48295.8 | 3 | 35.50% | 46362.8 | 3 | 34.80% | 0.96 | 54209.3 | 3 | 25.20% | 1.12 | 72020.6 | 3 | 30.50% | 1.49 |
| 24 | 41879.5 | 3 | 29.80% | 38753.4 | 3 | 27.80% | 0.93 | 50101.6 | 3 | 39.80% | 1.20 | 68726.4 | 3 | 19.80% | 1.64 |
| 25 | 85025.1 | 3 | 21.60% | 75964 | 3 | 26.60% | 0.89 | 60602.1 | 3 | 11.60% | 0.71 | 40602.1 | 3 | 21.80% | 0.48 |
| 26 | 50353.8 | 3 | 26.80% | 65381.2 | 3 | 29.80% | 1.30 | 77201.4 | 3 | 16.80% | 1.53 | 88412.7 | 3 | 20.80% | 1.76 |
| 27 | 43992.3 | 3 | 31.40% | 31426.6 | 3 | 21.80% | 0.71 | 45595.8 | 3 | 21.20% | 1.04 | 55606.9 | 3 | 38.50% | 1.26 |
| 28 | 58749.6 | 3 | 34.10% | 55656.7 | 3 | 36.80% | 0.95 | 70927 | 3 | 14.10% | 1.21 | 60312.4 | 3 | 30.20% | 1.03 |
| 29 | 238225.9 | 3 | 17.90% | 236330.8 | 3 | 27.80% | 0.99 | 205640.6 | 3 | 27.30% | 0.86 | 53125.8 | 3 | 15.60% | 0.22 |
| 30 | 88175.5 | 3 | 25.70% | 87004.3 | 3 | 25.70% | 0.99 | 77127.1 | 3 | 23.30% | 0.87 | 46682.1 | 3 | 21.40% | 0.53 |
| 31 | 99175.5 | 3 | 18.70% | 178004.3 | 3 | 18.70% | 1.79 | 137127.1 | 3 | 28.30% | 1.38 | 126682.1 | 3 | 13.70% | 1.28 |
| **Spot no** | **Control**  **(a)** | **N**  **(b)** | **CV**  **(c)** | **3 dpi**  **(a)** | **N**  **(b)** | **CV**  **(c)** | **Ratio**  **(d)** | **7 dpi**  **(a)** | **N**  **(b)** | **CV**  **(c)** | **Ratio**  **(d)** | **14 dpi**  **(a)** | **N**  **(b)** | **CV**  **(c)** | **Ratio**  **(d)** |
| 32 | 99130.8 | 3 | 32.10% | 56920.2 | 3 | 32.10% | 0.57 | 108851.7 | 3 | 33.20% | 1.10 | 65948.5 | 3 | 22.10% | 0.67 |
| 33 | 58836 | 3 | 35.10% | 49662.2 | 3 | 25.10% | 0.84 | 22427.5 | 3 | 25.10% | 0.38 | 19810.5 | 3 | 15.10% | 0.34 |
| 34 | 56666.9 | 3 | 36.10% | 69645.4 | 3 | 37.30% | 1.23 | 75022.5 | 3 | 37.10% | 1.32 | 85659 | 3 | 26.10% | 1.51 |
| 35 | 26354.9 | 3 | 19.10% | 37327.4 | 3 | 29.10% | 1.42 | 44701.5 | 3 | 29.10% | 1.70 | 58204.1 | 3 | 12.10% | 2.21 |
| 36 | 103636.9 | 3 | 22.80% | 100892.9 | 3 | 32.50% | 0.97 | 67039 | 3 | 21.80% | 0.65 | 56561.2 | 3 | 20.80% | 0.55 |
| 37 | 80719.9 | 3 | 19.70% | 104298.3 | 3 | 29.60% | 1.29 | 117700.6 | 3 | 11.70% | 1.46 | 52029.2 | 3 | 9.10% | 0.64 |
| 38 | 48836 | 3 | 15.60% | 39662.2 | 3 | 25.50% | 0.81 | 52427.5 | 3 | 12.60% | 1.07 | 66810.5 | 3 | 25.20% | 1.37 |
| 39 | 93581.9 | 3 | 24.50% | 58457.1 | 3 | 23.70% | 0.62 | 56977.6 | 3 | 21.50% | 0.61 | 52935.5 | 3 | 21.10% | 0.57 |
| 40 | 15743.4 | 3 | 25.10% | 20953.8 | 3 | 35.10% | 1.33 | 13699.7 | 3 | 15.20% | 0.87 | 26512.8 | 3 | 26.10% | 1.68 |
| 41 | 67520.3 | 3 | 21.00% | 53140.2 | 3 | 22.80% | 0.79 | 46683.3 | 3 | 22.80% | 0.69 | 70484.2 | 3 | 21.90% | 1.04 |
| 42 | 57768.4 | 3 | 30.70% | 60154.8 | 3 | 20.00% | 1.04 | 63926.2 | 3 | 20.70% | 1.11 | 69150.6 | 3 | 20.70% | 1.20 |
| 43 | 30166.2 | 3 | 26.70% | 39873.7 | 3 | 24.60% | 1.32 | 42669.2 | 3 | 22.70% | 1.41 | 20829 | 3 | 22.50% | 0.69 |
| 44 | 133875.7 | 3 | 24.40% | 219950.1 | 3 | 22.30% | 1.64 | 174592.1 | 3 | 22.10% | 1.30 | 107225.4 | 3 | 21.20% | 0.80 |
| 45 | 47768.4 | 3 | 20.80% | 30154.8 | 3 | 22.80% | 0.63 | 53926.2 | 3 | 21.80% | 1.13 | 66150.6 | 3 | 26.80% | 1.38 |
| 46 | 60166.2 | 3 | 37.40% | 29873.7 | 3 | 32.20% | 0.50 | 45669.2 | 3 | 27.40% | 0.76 | 80829 | 3 | 37.90% | 1.34 |
| 47 | 36552.7 | 3 | 26.40% | 35555.8 | 3 | 36.40% | 0.97 | 47221.7 | 3 | 21.40% | 1.29 | 43454.9 | 3 | 16.40% | 1.19 |
| 48 | 60663.6 | 3 | 18.60% | 58694.7 | 3 | 28.60% | 0.97 | 62555.3 | 3 | 28.10% | 1.03 | 67940.2 | 3 | 18.70% | 1.12 |
| **Spot no** | **Control**  **(a)** | **N**  **(b)** | **CV**  **(c)** | **3 dpi**  **(a)** | **N**  **(b)** | **CV**  **(c)** | **Ratio**  **(d)** | **7 dpi**  **(a)** | **N**  **(b)** | **CV**  **(c)** | **Ratio**  **(d)** | **14 dpi**  **(a)** | **N**  **(b)** | **CV**  **(c)** | **Ratio**  **(d)** |
| 49 | 44320 | 3 | 34.50% | 68969.9 | 3 | 33.50% | 1.56 | 67995.6 | 3 | 24.50% | 1.53 | 69962.3 | 3 | 24.50% | 1.58 |
| 50 | 41739.1 | 3 | 20.80% | 45762.7 | 3 | 22.80% | 1.10 | 47849.3 | 3 | 30.80% | 1.15 | 50847.2 | 3 | 22.80% | 1.22 |
| 51 | 30276.4 | 3 | 40.50% | 40360.9 | 3 | 30.50% | 1.33 | 54644.4 | 3 | 39.30% | 1.80 | 68042.6 | 3 | 30.90% | 2.25 |
| 52 | 34566.5 | 3 | 22.10% | 38018.9 | 3 | 21.10% | 1.10 | 41675 | 3 | 12.10% | 1.21 | 52425.5 | 3 | 23.30% | 1.52 |
| 53 | 49308.9 | 3 | 16.30% | 50660.5 | 3 | 26.30% | 1.03 | 33657.1 | 3 | 26.30% | 0.68 | 65277.1 | 3 | 26.90% | 1.32 |
| 54 | 34566.5 | 3 | 26.50% | 37018.9 | 3 | 24.30% | 1.07 | 40675 | 3 | 21.50% | 1.18 | 71425.5 | 3 | 24.10% | 2.07 |
| 55 | 50107.3 | 3 | 24.60% | 53876.1 | 3 | 34.60% | 1.08 | 64460.2 | 3 | 22.60% | 1.29 | 104943 | 3 | 29.60% | 2.09 |
| 56 | 46755 | 3 | 32.90% | 45672.3 | 3 | 33.90% | 0.98 | 44709.2 | 3 | 36.90% | 0.96 | 41765.3 | 3 | 32.90% | 0.89 |
| 57 | 46856.6 | 3 | 25.80% | 59717.7 | 3 | 35.60% | 1.27 | 60704.5 | 3 | 35.80% | 1.30 | 93348.3 | 3 | 35.80% | 1.99 |
| 58 | 44411.8 | 3 | 28.50% | 48457.5 | 3 | 18.40% | 1.09 | 58420.6 | 3 | 28.20% | 1.32 | 48420.6 | 3 | 27.50% | 1.09 |
| 59 | 41208.6 | 3 | 32.60% | 20467.1 | 3 | 22.60% | 0.50 | 52728.8 | 3 | 22.80% | 1.28 | 54126.1 | 3 | 12.60% | 1.31 |
| 60 | 56552.7 | 3 | 27.50% | 35555.8 | 3 | 21.50% | 0.63 | 57221.7 | 3 | 23.10% | 1.01 | 85454.9 | 3 | 20.50% | 1.51 |
| 61 | 68561.7 | 3 | 39.80% | 73999.7 | 3 | 29.80% | 1.08 | 86805.1 | 3 | 38.80% | 1.27 | 95597 | 3 | 19.80% | 1.39 |
| 62 | 75019.6 | 3 | 32.10% | 79210.7 | 3 | 12.60% | 1.06 | 86524.2 | 3 | 32.00% | 1.15 | 97983.3 | 3 | 12.10% | 1.31 |
| 63 | 49821.7 | 3 | 36.70% | 28429.9 | 3 | 26.50% | 0.57 | 18668.1 | 3 | 31.60% | 0.37 | 14323 | 3 | 30.70% | 0.29 |
| 64 | 49822 | 3 | 34.60% | 50408 | 3 | 24.60% | 1.01 | 68852.7 | 3 | 24.40% | 1.38 | 71253.7 | 3 | 26.60% | 1.43 |
| 65 | 38856.6 | 3 | 19.60% | 19717.7 | 3 | 39.70% | 0.51 | 40704.5 | 3 | 29.20% | 1.05 | 63348.3 | 3 | 29.60% | 1.63 |
| Spot no | Control  (a) | N  (b) | CV  (c) | 3 dpi  (a) | N  (b) | CV  (c) | Ratio  (d) | 7 dpi  (a) | N  (b) | CV  (c) | Ratio  (d) | 14 dpi  (a) | N  (b) | CV  (c) | Ratio  (d) |
| 66 | 12316 | 3 | 26.40% | 12656 | 3 | 24.40% | 1.03 | 11876 | 3 | 36.10% | 0.96 | 10987 | 3 | 21.40% | 0.89 |
| 67 | 21342 | 3 | 29.60% | 23416 | 3 | 25.60% | 1.10 | 20987 | 3 | 19.60% | 0.98 | 18765 | 3 | 24.60% | 0.88 |
| 68 | 19876 | 3 | 30.50% | 17865 | 3 | 20.50% | 0.90 | 16564 | 3 | 20.50% | 0.83 | 14326 | 3 | 20.50% | 0.72 |
| 69 | 44822 | 3 | 27.10% | 30408 | 3 | 27.10% | 0.68 | 48852.7 | 3 | 37.20% | 1.09 | 59253.7 | 3 | 24.10% | 1.32 |
| 70 | 49655 | 3 | 31.80% | 46012.3 | 3 | 21.80% | 0.93 | 50709.2 | 3 | 21.50% | 1.02 | 67285.3 | 3 | 21.80% | 1.36 |
| 71 | 19124.6 | 3 | 12.00% | 12424.8 | 3 | 12.90% | 0.65 | 14509.9 | 3 | 22.00% | 0.76 | 29533.2 | 3 | 12.87% | 1.54 |
| 72 | 3241 | 3 | 6.10% | 3432 | 3 | 16.10% | 1.06 | 2865 | 3 | 16.10% | 0.88 | 2765 | 3 | 16.10% | 0.85 |
| 73 | 23765.4 | 3 | 34.10% | 15487.9 | 3 | 14.10% | 0.65 | 13241 | 3 | 14.20% | 0.56 | 11875 | 3 | 14.10% | 0.50 |
| 74 | 23411 | 3 | 38.60% | 22341 | 3 | 30.60% | 0.95 | 19876 | 3 | 28.60% | 0.85 | 18765 | 3 | 32.60% | 0.80 |
| 75 | 21321 | 3 | 29.20% | 20987 | 3 | 21.40% | 0.98 | 18765 | 3 | 26.20% | 0.88 | 17654 | 3 | 24.20% | 0.83 |
| 76 | 43251 | 3 | 33.60% | 41231 | 3 | 32.60% | 0.95 | 38761 | 3 | 13.60% | 0.90 | 40987 | 3 | 23.60% | 0.95 |
| 77 | 4321 | 3 | 34.00% | 3876 | 3 | 34.80% | 0.90 | 3452 | 3 | 24.32% | 0.80 | 3821 | 3 | 14.90% | 0.88 |
| 78 | 2314 | 3 | 23.20% | 2654 | 3 | 13.20% | 1.15 | 2876 | 3 | 13.20% | 1.24 | 2132 | 3 | 13.20% | 0.92 |
| 79 | 98561.7 | 3 | 30.20% | 33999.7 | 3 | 31.20% | 0.34 | 36805.1 | 3 | 20.20% | 0.37 | 96597 | 3 | 20.20% | 0.98 |
| 80 | 34213 | 3 | 30.90% | 28764 | 3 | 20.90% | 0.84 | 26512 | 3 | 32.90% | 0.77 | 25432 | 3 | 32.90% | 0.74 |
| 81 | 55907.5 | 3 | 26.50% | 48343.3 | 3 | 25.40% | 0.86 | 57822.1 | 3 | 22.50% | 1.03 | 74564.3 | 3 | 28.00% | 1.33 |
| 82 | 23125 | 3 | 23.70% | 21654 | 3 | 24.70% | 0.94 | 24521 | 3 | 21.40% | 1.06 | 27654 | 3 | 21.70% | 1.20 |
| **Spot no** | **Control**  **(a)** | **N**  **(b)** | **CV**  **(c)** | **3 dpi**  **(a)** | **N**  **(b)** | **CV**  **(c)** | **Ratio**  **(d)** | **7 dpi**  **(a)** | **N**  **(b)** | **CV**  **(c)** | **Ratio**  **(d)** | **14 dpi**  **(a)** | **N**  **(b)** | **CV**  **(c)** | **Ratio**  **(d)** |
| 83 | 99848.2 | 3 | 25.30% | 44765.3 | 3 | 22.30% | 0.45 | 61433.1 | 3 | 22.10% | 0.62 | 112752.8 | 3 | 22.30% | 1.13 |
| 84 | 36727.9 | 3 | 29.80% | 16475.3 | 3 | 26.50% | 0.45 | 27732.6 | 3 | 22.80% | 0.76 | 68034.9 | 3 | 21.20% | 1.85 |
| 85 | 11214 | 3 | 26.32% | 12123 | 3 | 26.00% | 1.08 | 10987 | 3 | 22.32% | 0.98 | 13241 | 3 | 21.30% | 1.18 |
| 86 | 10987 | 3 | 21.54% | 9876 | 3 | 20.50% | 0.90 | 11765 | 3 | 23.14% | 1.07 | 10765 | 3 | 11.54% | 0.98 |
| 87 | 12171 | 3 | 32.12% | 10987 | 3 | 22.12% | 0.90 | 13214 | 3 | 22.12% | 1.09 | 12675 | 3 | 39.12% | 1.04 |
| 88 | 16543 | 3 | 28.30% | 15432 | 3 | 23.10% | 0.93 | 14325 | 3 | 21.30% | 0.87 | 12326 | 3 | 18.30% | 0.75 |
| 89 | 38274.4 | 3 | 36.30% | 19796.8 | 3 | 32.10% | 0.52 | 24928.8 | 3 | 16.30% | 0.65 | 21732.1 | 3 | 36.90% | 0.57 |
| 90 | 21212 | 3 | 31.70% | 19874 | 3 | 11.50% | 0.94 | 20987 | 3 | 21.20% | 0.99 | 18765 | 3 | 21.60% | 0.88 |
| 91 | 12316 | 3 | 23.00% | 14213 | 3 | 22.00% | 1.15 | 11986 | 3 | 13.20% | 0.97 | 12154 | 3 | 21.00% | 0.99 |
| 92 | 12342 | 3 | 32.20% | 11254 | 3 | 22.20% | 0.91 | 13216 | 3 | 22.20% | 1.07 | 10987 | 3 | 12.20% | 0.89 |
| 93 | 9923.3 | 3 | 11.70% | 11274 | 3 | 21.70% | 1.14 | 12435 | 3 | 21.70% | 1.25 | 13241 | 3 | 11.10% | 1.33 |
| 94 | 30824 | 3 | 15.87% | 33860 | 3 | 14.57% | 1.10 | 29670 | 3 | 15.20% | 0.96 | 26457 | 3 | 19.87% | 0.86 |
| 95 | 28274.4 | 3 | 27.98% | 21796.8 | 3 | 25.78% | 0.77 | 24928.8 | 3 | 17.98% | 0.88 | 33173.1 | 3 | 17.98% | 1.17 |
| 96 | 22242.7 | 3 | 29.65% | 15478.3 | 3 | 26.65% | 0.70 | 13425 | 3 | 29.00% | 0.60 | 16543 | 3 | 28.50% | 0.74 |
| 97 | 17994.2 | 3 | 21.97% | 16391.3 | 3 | 20.97% | 0.91 | 23619.8 | 3 | 21.90% | 1.31 | 20619.8 | 3 | 11.97% | 1.15 |
| 98 | 42082.8 | 3 | 28.96% | 34206.4 | 3 | 22.91% | 0.81 | 59391.8 | 3 | 21.91% | 1.41 | 61119.1 | 3 | 20.96% | 1.45 |
| 99 | 32082.8 | 3 | 26.80% | 64206.4 | 3 | 24.70% | 2.00 | 49391.8 | 3 | 21.80% | 1.54 | 79119.1 | 3 | 25.00% | 2.47 |
| **Spot no** | **Control**  **(a)** | **N**  **(b)** | **CV**  **(c)** | **3 dpi**  **(a)** | **N**  **(b)** | **CV**  **(c)** | **Ratio**  **(d)** | **7 dpi**  **(a)** | **N**  **(b)** | **CV**  **(c)** | **Ratio**  **(d)** | **14 dpi**  **(a)** | **N**  **(b)** | **CV**  **(c)** | **Ratio**  **(d)** |
| 100 | 30124 | 3 | 35.70% | 31860 | 3 | 15.60% | 1.06 | 36124 | 3 | 15.30% | 1.20 | 40860 | 3 | 15.70% | 1.36 |
| 101 | 21124 | 3 | 19.60% | 24860 | 3 | 17.20% | 1.18 | 22670 | 3 | 12.60% | 1.07 | 21127 | 3 | 39.60% | 1.00 |
| 102 | 22124 | 3 | 27.60% | 25360 | 3 | 24.40% | 1.15 | 20670 | 3 | 21.10% | 0.93 | 20217 | 3 | 28.60% | 0.91 |
| 103 | 80831.4 | 3 | 38.00% | 65145.7 | 3 | 28.00% | 0.81 | 65518 | 3 | 36.90% | 0.81 | 78456.3 | 3 | 18.00% | 0.97 |
| 104 | 38280.9 | 3 | 20.80% | 39766.6 | 3 | 22.50% | 1.04 | 44264.7 | 3 | 22.80% | 1.16 | 65496 | 3 | 22.80% | 1.71 |
| 105 | 32242.7 | 3 | 32.30% | 16378.3 | 3 | 22.30% | 0.51 | 13425 | 3 | 22.30% | 0.42 | 11564 | 3 | 12.30% | 0.36 |
| 106 | 19043.7 | 3 | 30.70% | 17057.2 | 3 | 20.70% | 0.90 | 7681.7 | 3 | 31.70% | 0.40 | 6578 | 3 | 20.20% | 0.35 |
| 107 | 31124 | 3 | 22.00% | 28860 | 3 | 12.00% | 0.93 | 25670 | 3 | 21.20% | 0.82 | 21457 | 3 | 29.20% | 0.69 |
| 108 | 191743.3 | 3 | 32.20% | 216724 | 3 | 22.20% | 1.13 | 208866.5 | 3 | 12.20% | 1.09 | 118339.3 | 3 | 12.20% | 0.62 |
| 109 | 33124 | 3 | 12.70% | 36860 | 3 | 11.70% | 1.11 | 31124 | 3 | 22.90% | 0.94 | 40860 | 3 | 16.70% | 1.23 |
| **Incompatible interaction** | | | | | | | | | | | | | | | |
| 1 | 1022386 | 3 | 21.70% | 1371508.3 | 3 | 20.80% | 1.34 | 2039884.3 | 3 | 19.80% | 2.00 | 1942663 | 3 | 10.47% | 1.90 |
| 2 | 1401274.8 | 3 | 20.00% | 1563116.5 | 3 | 14.30% | 1.12 | 1885780.1 | 3 | 22.80% | 1.35 | 2345678 | 3 | 24.21% | 1.67 |
| 3 | 2983104.5 | 3 | 26.70% | 3402457.5 | 3 | 35.60% | 1.14 | 4737248.4 | 3 | 25.79% | 1.59 | 5884769.8 | 3 | 25.70% | 1.97 |
| 4 | 1493882.6 | 3 | 27.80% | 5868087.5 | 3 | 31.30% | 3.93 | 4055274.9 | 3 | 29.80% | 2.71 | 4194257.5 | 3 | 21.34% | 2.81 |
| 5 | 279664.8 | 3 | 23.30% | 308883.9 | 3 | 28.30% | 1.10 | 464309.5 | 3 | 38.40% | 1.66 | 456153 | 3 | 18.13% | 1.63 |
| 6 | 291196.3 | 3 | 32.24% | 559322.3 | 3 | 22.34% | 1.92 | 452873.7 | 3 | 31.24% | 1.56 | 383775.1 | 3 | 11.34% | 1.32 |
| **Spot no** | **Control**  **(a)** | **N**  **(b)** | **CV**  **(c)** | **3 dpi**  **(a)** | **N**  **(b)** | **CV**  **(c)** | **Ratio**  **(d)** | **7 dpi**  **(a)** | **N**  **(b)** | **CV**  **(c)** | **Ratio**  **(d)** | **14 dpi**  **(a)** | **N**  **(b)** | **CV**  **(c)** | **Ratio**  **(d)** |
| 7 | 465846.7 | 3 | 32.50% | 592776.6 | 3 | 22.50% | 1.27 | 693604.9 | 3 | 39.50% | 1.49 | 654887.1 | 3 | 22.15% | 1.41 |
| 8 | 311691.5 | 3 | 31.10% | 397178.5 | 3 | 39.50% | 1.27 | 415701 | 3 | 36.15% | 1.33 | 439421.1 | 3 | 39.45% | 1.41 |
| 9 | 557309.5 | 3 | 31.90% | 567909.5 | 3 | 22.70% | 1.02 | 587309.5 | 3 | 34.90% | 1.05 | 594309.5 | 3 | 37.19% | 1.07 |
| 10 | 1098777.5 | 3 | 26.60% | 974386 | 3 | 22.70% | 0.89 | 841979.6 | 3 | 37.60% | 0.77 | 815739.7 | 3 | 35.16% | 0.74 |
| 11 | 487309.5 | 3 | 32.50% | 565477.4 | 3 | 21.30% | 1.16 | 625774.6 | 3 | 30.30% | 1.28 | 677396 | 3 | 20.23% | 1.39 |
| 12 | 235259 | 3 | 29.20% | 315521.8 | 3 | 29.50% | 1.34 | 358428.4 | 3 | 25.70% | 1.52 | 387052 | 3 | 19.57% | 1.65 |
| 13 | 896296.1 | 3 | 21.60% | 922180.8 | 3 | 22.30% | 1.03 | 1238093.1 | 3 | 35.30% | 1.38 | 1247468.5 | 3 | 25.33% | 1.39 |
| 14 | 1195031 | 3 | 21.80% | 1244564.9 | 3 | 40.80% | 1.04 | 1444564.9 | 3 | 21.80% | 1.21 | 1544564.9 | 3 | 31.38% | 1.29 |
| 15 | 1106147.1 | 3 | 25.60% | 999534.4 | 3 | 21.40% | 0.90 | 1117628.7 | 3 | 36.40% | 1.01 | 1163849.2 | 3 | 27.54% | 1.05 |
| 16 | 107461.1 | 3 | 36.80% | 142639.6 | 3 | 24.60% | 1.33 | 192659.6 | 3 | 37.20% | 1.79 | 159796.5 | 3 | 23.46% | 1.49 |
| 17 | 310845.9 | 3 | 33.60% | 360971.3 | 3 | 21.40% | 1.16 | 517599.4 | 3 | 34.44% | 1.67 | 453424.3 | 3 | 37.56% | 1.46 |
| 18 | 266488.8 | 3 | 21.60% | 368847.5 | 3 | 20.80% | 1.38 | 640225.7 | 3 | 23.63% | 2.40 | 667801.6 | 3 | 23.56% | 2.51 |
| 19 | 408253.1 | 3 | 38.60% | 420912.3 | 3 | 15.50% | 1.03 | 547215.1 | 3 | 28.51% | 1.34 | 590171.9 | 3 | 18.55% | 1.45 |
| 20 | 179004.8 | 3 | 21.70% | 296212.8 | 3 | 21.80% | 1.65 | 525577.7 | 3 | 22.28% | 2.94 | 502143.8 | 3 | 22.58% | 2.81 |
| 21 | 1550647.5 | 3 | 16.70% | 1617017.3 | 3 | 27.80% | 1.04 | 1959957.3 | 3 | 37.71% | 1.26 | 1729532.6 | 3 | 21.47% | 1.12 |
| 22 | 203376.8 | 3 | 31.70% | 238622.3 | 3 | 31.90% | 1.17 | 126462 | 3 | 34.91% | 0.62 | 88921.9 | 3 | 34.90% | 0.44 |
| 23 | 75886.6 | 3 | 31.80% | 85378.6 | 3 | 33.50% | 1.13 | 89988.1 | 3 | 25.52% | 1.19 | 98801.4 | 3 | 15.25% | 1.30 |
| **Spot no** | **Control**  **(a)** | **N**  **(b)** | **CV**  **(c)** | **3 dpi**  **(a)** | **N**  **(b)** | **CV**  **(c)** | **Ratio**  **(d)** | **7 dpi**  **(a)** | **N**  **(b)** | **CV**  **(c)** | **Ratio**  **(d)** | **14 dpi**  **(a)** | **N**  **(b)** | **CV**  **(c)** | **Ratio**  **(d)** |
| 24 | 246739.4 | 3 | 24.80% | 329053 | 3 | 29.80% | 1.33 | 376064.8 | 3 | 19.85% | 1.52 | 332448.7 | 3 | 19.58% | 1.35 |
| 25 | 142722.4 | 3 | 16.60% | 182894.6 | 3 | 21.50% | 1.28 | 274906.9 | 3 | 28.60% | 1.93 | 287920.1 | 3 | 11.64% | 2.02 |
| 26 | 104518.3 | 3 | 28.80% | 71320.6 | 3 | 22.80% | 0.68 | 66558.8 | 3 | 36.87% | 0.64 | 109292.7 | 3 | 23.58% | 1.05 |
| 27 | 61504.9 | 3 | 22.80% | 70243.6 | 3 | 35.50% | 1.14 | 134800.9 | 3 | 21.45% | 2.19 | 130133.2 | 3 | 21.40% | 2.12 |
| 28 | 227048.1 | 3 | 32.80% | 271291.4 | 3 | 33.70% | 1.19 | 354153.8 | 3 | 40.10% | 1.56 | 276310.3 | 3 | 24.10% | 1.22 |
| 29 | 395157.8 | 3 | 17.80% | 571511.4 | 3 | 25.60% | 1.45 | 473487.1 | 3 | 27.91% | 1.20 | 450200.4 | 3 | 15.90% | 1.14 |
| 30 | 246034.8 | 3 | 24.70% | 303047.5 | 3 | 28.40% | 1.23 | 280199.2 | 3 | 15.79% | 1.14 | 253858.5 | 3 | 22.37% | 1.03 |
| 31 | 315127.6 | 3 | 18.70% | 684560.4 | 3 | 13.90% | 2.17 | 723689.1 | 3 | 28.79% | 2.30 | 643715.4 | 3 | 28.70% | 2.04 |
| 32 | 245563.8 | 3 | 32.80% | 338426.4 | 3 | 21.10% | 1.38 | 393835.5 | 3 | 22.16% | 1.60 | 288214.2 | 3 | 31.10% | 1.17 |
| 33 | 817437.6 | 3 | 21.70% | 898015.5 | 3 | 35.10% | 1.10 | 671058.2 | 3 | 25.18% | 0.82 | 628068.7 | 3 | 30.41% | 0.77 |
| 34 | 593693.1 | 3 | 27.30% | 953021.4 | 3 | 24.90% | 1.61 | 855441.6 | 3 | 26.31% | 1.44 | 829057.8 | 3 | 26.10% | 1.40 |
| 35 | 95129 | 3 | 23.10% | 218118.4 | 3 | 22.90% | 2.29 | 149533.9 | 3 | 29.15% | 1.57 | 171223.7 | 3 | 29.10% | 1.80 |
| 36 | 65848.4 | 3 | 32.80% | 85848.4 | 3 | 23.80% | 1.30 | 99943.7 | 3 | 32.80% | 1.52 | 85181.9 | 3 | 20.80% | 1.29 |
| 37 | 718355.6 | 3 | 23.60% | 684745 | 3 | 19.10% | 0.95 | 296143.6 | 3 | 29.70% | 0.41 | 266111.5 | 3 | 39.70% | 0.37 |
| 38 | 332217.8 | 3 | 35.50% | 602789.2 | 3 | 21.20% | 1.81 | 695184.7 | 3 | 25.60% | 2.09 | 685249.2 | 3 | 25.46% | 2.06 |
| 39 | 541705 | 3 | 13.70% | 757255.5 | 3 | 22.10% | 1.40 | 955673.9 | 3 | 27.15% | 1.76 | 815184.1 | 3 | 21.25% | 1.50 |
| 40 | 203393.1 | 3 | 39.10% | 420786.9 | 3 | 25.50% | 2.07 | 360276.2 | 3 | 25.19% | 1.77 | 425198.3 | 3 | 22.10% | 2.09 |
| **Spot no** | **Control**  **(a)** | **N**  **(b)** | **CV**  **(c)** | **3 dpi**  **(a)** | **N**  **(b)** | **CV**  **(c)** | **Ratio**  **(d)** | **7 dpi**  **(a)** | **N**  **(b)** | **CV**  **(c)** | **Ratio**  **(d)** | **14 dpi**  **(a)** | **N**  **(b)** | **CV**  **(c)** | **Ratio**  **(d)** |
| 41 | 364732.5 | 3 | 12.80% | 418211.5 | 3 | 29.70% | 1.15 | 382795.5 | 3 | 11.40% | 1.05 | 397071.4 | 3 | 21.23% | 1.09 |
| 42 | 98504.2 | 3 | 20.90% | 65689.1 | 3 | 22.90% | 0.67 | 53923.2 | 3 | 34.70% | 0.55 | 53449.4 | 3 | 25.70% | 0.54 |
| 43 | 96723.5 | 3 | 14.60% | 101329.7 | 3 | 29.90% | 1.05 | 133250.2 | 3 | 22.74% | 1.38 | 354104.8 | 3 | 22.57% | 3.66 |
| 44 | 896660.6 | 3 | 21.30% | 931519.1 | 3 | 23.72% | 1.04 | 1695750.5 | 3 | 21.42% | 1.89 | 1141811.3 | 3 | 20.44% | 1.27 |
| 45 | 94851.3 | 3 | 22.90% | 123357.5 | 3 | 22.81% | 1.30 | 143661.6 | 3 | 22.18% | 1.51 | 124770.7 | 3 | 25.18% | 1.32 |
| 46 | 204077.1 | 3 | 30.20% | 85588 | 3 | 33.90% | 0.42 | 88388.1 | 3 | 34.40% | 0.43 | 88234.5 | 3 | 27.44% | 0.43 |
| 47 | 194401.7 | 3 | 36.10% | 378565.8 | 3 | 26.40% | 1.95 | 332255.5 | 3 | 27.70% | 1.71 | 272094.4 | 3 | 21.40% | 1.40 |
| 48 | 191053.9 | 3 | 18.60% | 203051.4 | 3 | 28.70% | 1.06 | 668932.4 | 3 | 28.65% | 3.50 | 444310.4 | 3 | 38.60% | 2.33 |
| 49 | 228318.2 | 3 | 23.50% | 340938.2 | 3 | 34.50% | 1.49 | 544029.8 | 3 | 24.53% | 2.38 | 501935.1 | 3 | 24.45% | 2.20 |
| 50 | 179678 | 3 | 21.80% | 296199 | 3 | 29.70% | 1.65 | 648299.3 | 3 | 22.80% | 3.61 | 791645 | 3 | 22.80% | 4.41 |
| 51 | 504040 | 3 | 37.50% | 860503 | 3 | 32.90% | 1.71 | 875152.6 | 3 | 34.50% | 1.74 | 728953 | 3 | 20.50% | 1.45 |
| 52 | 193893.2 | 3 | 11.10% | 161498.4 | 3 | 27.30% | 0.83 | 471195.5 | 3 | 27.15% | 2.43 | 420876.7 | 3 | 20.10% | 2.17 |
| 53 | 11157 | 3 | 26.30% | 17369.6 | 3 | 25.90% | 1.56 | 27141.8 | 3 | 26.30% | 2.43 | 33351.9 | 3 | 36.53% | 2.99 |
| 54 | 107348.3 | 3 | 24.10% | 146552.3 | 3 | 21.10% | 1.37 | 189444.7 | 3 | 29.52% | 1.76 | 170341.8 | 3 | 16.52% | 1.59 |
| 55 | 311767.6 | 3 | 34.10% | 149550.9 | 3 | 39.60% | 0.48 | 170514.3 | 3 | 26.65% | 0.55 | 16608.1 | 3 | 21.26% | 0.05 |
| 56 | 32251.2 | 3 | 13.90% | 41570.9 | 3 | 40.90% | 1.29 | 60993.2 | 3 | 12.95% | 1.89 | 66030.7 | 3 | 22.39% | 2.05 |
| 57 | 152768.4 | 3 | 38.60% | 147743.6 | 3 | 25.80% | 0.97 | 139895.3 | 3 | 15.80% | 0.92 | 131691.8 | 3 | 20.80% | 0.86 |
| **Spot no** | **Control**  **(a)** | **N**  **(b)** | **CV**  **(c)** | **3 dpi**  **(a)** | **N**  **(b)** | **CV**  **(c)** | **Ratio**  **(d)** | **7 dpi**  **(a)** | **N**  **(b)** | **CV**  **(c)** | **Ratio**  **(d)** | **14 dpi**  **(a)** | **N**  **(b)** | **CV**  **(c)** | **Ratio**  **(d)** |
| 58 | 39251.2 | 3 | 17.40% | 61570.9 | 3 | 21.50% | 1.57 | 80993.2 | 3 | 18.50% | 2.06 | 96030.7 | 3 | 21.55% | 2.45 |
| 59 | 138606.1 | 3 | 22.90% | 339521.4 | 3 | 32.10% | 2.45 | 215344.5 | 3 | 22.16% | 1.55 | 302082.8 | 3 | 12.46% | 2.18 |
| 60 | 206754.9 | 3 | 20.50% | 304452.8 | 3 | 20.53% | 1.47 | 368841.6 | 3 | 37.50% | 1.78 | 311004 | 3 | 17.54% | 1.50 |
| 61 | 156708 | 3 | 29.90% | 180649.4 | 3 | 29.87% | 1.15 | 252763.2 | 3 | 33.80% | 1.61 | 225278.8 | 3 | 29.58% | 1.44 |
| 62 | 156708 | 3 | 22.60% | 170649.4 | 3 | 22.71% | 1.09 | 222763.2 | 3 | 12.50% | 1.42 | 215278.8 | 3 | 12.41% | 1.37 |
| 63 | 118690 | 3 | 24.50% | 147771.9 | 3 | 31.74% | 1.25 | 180163.4 | 3 | 26.57% | 1.52 | 195190.6 | 3 | 26.70% | 1.64 |
| 64 | 117690 | 3 | 24.10% | 142771.9 | 3 | 36.60% | 1.21 | 170163.4 | 3 | 36.60% | 1.45 | 165190.6 | 3 | 34.60% | 1.40 |
| 65 | 83405.6 | 3 | 29.70% | 93680.9 | 3 | 28.60% | 1.12 | 172729.1 | 3 | 15.60% | 2.07 | 162969.5 | 3 | 29.65% | 1.95 |
| 66 | 116690 | 3 | 34.40% | 123771.9 | 3 | 23.40% | 1.06 | 169163.4 | 3 | 16.40% | 1.45 | 135690.6 | 3 | 21.43% | 1.16 |
| 67 | 12504.8 | 3 | 28.60% | 12713.6 | 3 | 25.60% | 1.02 | 13562.2 | 3 | 25.60% | 1.08 | 13245.3 | 3 | 24.56% | 1.06 |
| 68 | 15207.5 | 3 | 29.50% | 16590 | 3 | 23.53% | 1.09 | 16032.5 | 3 | 31.59% | 1.05 | 14842.7 | 3 | 31.50% | 0.98 |
| 69 | 128690 | 3 | 24.10% | 167771.9 | 3 | 29.14% | 1.30 | 170163.4 | 3 | 17.21% | 1.32 | 175190.6 | 3 | 27.16% | 1.36 |
| 70 | 459074.3 | 3 | 31.80% | 599928.5 | 3 | 20.80% | 1.31 | 650281.1 | 3 | 21.58% | 1.42 | 702612 | 3 | 21.48% | 1.53 |
| 71 | 42500.8 | 3 | 32.90% | 65113.6 | 3 | 32.87% | 1.53 | 265626.2 | 3 | 12.43% | 6.25 | 288888.3 | 3 | 12.50% | 6.80 |
| 72 | 35607.5 | 3 | 26.10% | 45909 | 3 | 26.19% | 1.29 | 140326.5 | 3 | 16.10% | 3.94 | 137849.7 | 3 | 26.17% | 3.87 |
| 73 | 74625.2 | 3 | 24.10% | 88739.8 | 3 | 24.19% | 1.19 | 207734.2 | 3 | 24.13% | 2.78 | 191410.5 | 3 | 24.71% | 2.56 |
| 74 | 62719.3 | 3 | 32.60% | 65717 | 3 | 32.60% | 1.05 | 112728.9 | 3 | 28.26% | 1.80 | 71276.3 | 3 | 18.46% | 1.14 |
| **Spot no** | **Control**  **(a)** | **N**  **(b)** | **CV**  **(c)** | **3 dpi**  **(a)** | **N**  **(b)** | **CV**  **(c)** | **Ratio**  **(d)** | **7 dpi**  **(a)** | **N**  **(b)** | **CV**  **(c)** | **Ratio**  **(d)** | **14 dpi**  **(a)** | **N**  **(b)** | **CV**  **(c)** | **Ratio**  **(d)** |
| 75 | 61919.3 | 3 | 31.40% | 65717 | 3 | 20.20% | 1.06 | 72728.9 | 3 | 21.12% | 1.17 | 71276.3 | 3 | 22.52% | 1.15 |
| 76 | 81743.6 | 3 | 30.60% | 98011.5 | 3 | 29.67% | 1.20 | 87105.2 | 3 | 23.56% | 1.07 | 88068.7 | 3 | 23.56% | 1.08 |
| 77 | 61793.6 | 3 | 34.10% | 68011.5 | 3 | 34.92% | 1.10 | 97105.2 | 3 | 24.34% | 1.57 | 88765.7 | 3 | 24.00% | 1.44 |
| 78 | 88643.6 | 3 | 11.20% | 89651.5 | 3 | 23.20% | 1.01 | 107105.2 | 3 | 21.20% | 1.21 | 118068.7 | 3 | 21.52% | 1.33 |
| 79 | 129958 | 3 | 37.20% | 143178.2 | 3 | 22.20% | 1.10 | 159768.5 | 3 | 32.42% | 1.23 | 180291.1 | 3 | 20.20% | 1.39 |
| 80 | 129958 | 3 | 27.90% | 133198.2 | 3 | 31.99% | 1.02 | 149768.5 | 3 | 20.19% | 1.15 | 150691.1 | 3 | 38.90% | 1.16 |
| 81 | 193334.5 | 3 | 35.40% | 226491.1 | 3 | 28.97% | 1.17 | 411790.5 | 3 | 16.20% | 2.13 | 259195.6 | 3 | 16.50% | 1.34 |
| 82 | 139148.3 | 3 | 34.70% | 169836 | 3 | 24.70% | 1.22 | 374497.3 | 3 | 13.57% | 2.69 | 346497.3 | 3 | 26.70% | 2.49 |
| 83 | 320005 | 3 | 32.30% | 495552.2 | 3 | 27.30% | 1.55 | 663831.4 | 3 | 28.30% | 2.07 | 763539.6 | 3 | 26.30% | 2.39 |
| 84 | 119958 | 3 | 29.50% | 153178.2 | 3 | 27.20% | 1.28 | 179768.5 | 3 | 22.60% | 1.50 | 190291.1 | 3 | 27.48% | 1.59 |
| 85 | 123458 | 3 | 26.97% | 167897.2 | 3 | 31.30% | 1.36 | 223468.5 | 3 | 25.20% | 1.81 | 200291.1 | 3 | 21.32% | 1.62 |
| 86 | 124258 | 3 | 22.50% | 167658.2 | 3 | 21.54% | 1.35 | 197876.5 | 3 | 22.40% | 1.59 | 184561.1 | 3 | 25.40% | 1.49 |
| 87 | 229958 | 3 | 32.12% | 231278.2 | 3 | 38.19% | 1.01 | 241276.5 | 3 | 33.20% | 1.05 | 219861.1 | 3 | 22.12% | 0.96 |
| 88 | 176958 | 3 | 29.10% | 187678.2 | 3 | 28.30% | 1.06 | 177321.5 | 3 | 25.30% | 1.00 | 190765.1 | 3 | 24.33% | 1.08 |
| 89 | 116161.1 | 3 | 33.10% | 170510.8 | 3 | 26.99% | 1.47 | 198485.1 | 3 | 30.13% | 1.71 | 137251.8 | 3 | 31.30% | 1.18 |
| 90 | 32912.3 | 3 | 31.50% | 15824.9 | 3 | 28.60% | 0.48 | 27651.8 | 3 | 11.57% | 0.84 | 32254 | 3 | 21.57% | 0.98 |
| 91 | 3213.3 | 3 | 23.90% | 3124.9 | 3 | 21.92% | 0.97 | 3214.8 | 3 | 21.00% | 1.00 | 3325 | 3 | 27.90% | 1.03 |
| **Spot no** | **Control**  **(a)** | **N**  **(b)** | **CV**  **(c)** | **3 dpi**  **(a)** | **N**  **(b)** | **CV**  **(c)** | **Ratio**  **(d)** | **7 dpi**  **(a)** | **N**  **(b)** | **CV**  **(c)** | **Ratio**  **(d)** | **14 dpi**  **(a)** | **N**  **(b)** | **CV**  **(c)** | **Ratio**  **(d)** |
| 92 | 52903.3 | 3 | 27.20% | 51241.9 | 3 | 22.20% | 0.97 | 46175.8 | 3 | 12.20% | 0.87 | 52256 | 3 | 31.12% | 0.99 |
| 93 | 58811.2 | 3 | 25.70% | 71958.1 | 3 | 31.19% | 1.22 | 123111.4 | 3 | 21.30% | 2.09 | 112609.8 | 3 | 15.27% | 1.91 |
| 94 | 4213.3 | 3 | 24.57% | 3824.9 | 3 | 17.87% | 0.91 | 3214.8 | 3 | 19.70% | 0.76 | 3725 | 3 | 25.87% | 0.88 |
| 95 | 129148.3 | 3 | 35.78% | 149836 | 3 | 27.98% | 1.16 | 356497.3 | 3 | 25.80% | 2.76 | 366497.3 | 3 | 17.85% | 2.84 |
| 96 | 133910.1 | 3 | 26.80% | 225457.1 | 3 | 38.50% | 1.68 | 263657 | 3 | 21.50% | 1.97 | 192505.6 | 3 | 27.50% | 1.44 |
| 97 | 62903.3 | 3 | 22.97% | 58214.9 | 3 | 21.97% | 0.93 | 46175.8 | 3 | 20.70% | 0.73 | 52256 | 3 | 22.70% | 0.83 |
| 98 | 262957 | 3 | 32.91% | 250942.5 | 3 | 23.96% | 0.95 | 223255.7 | 3 | 18.60% | 0.85 | 246404.6 | 3 | 21.60% | 0.94 |
| 99 | 249905.6 | 3 | 34.70% | 269853.7 | 3 | 25.98% | 1.08 | 323279.1 | 3 | 24.18% | 1.29 | 343050.8 | 3 | 20.80% | 1.37 |
| 100 | 64173.7 | 3 | 25.60% | 86790.3 | 3 | 35.70% | 1.35 | 82666.6 | 3 | 25.17% | 1.29 | 84025.6 | 3 | 15.67% | 1.31 |
| 101 | 58173.7 | 3 | 37.20% | 86790.3 | 3 | 35.64% | 1.49 | 92666.6 | 3 | 12.16% | 1.59 | 94025.6 | 3 | 29.30% | 1.62 |
| 102 | 223542.5 | 3 | 34.40% | 255678.9 | 3 | 38.60% | 1.14 | 285870 | 3 | 25.16% | 1.28 | 228583.3 | 3 | 22.56% | 1.02 |
| 103 | 146589.1 | 3 | 28.87% | 161362.9 | 3 | 28.78% | 1.10 | 205443.8 | 3 | 28.20% | 1.40 | 192483.2 | 3 | 31.20% | 1.31 |
| 104 | 276720.5 | 3 | 34.50% | 356330.9 | 3 | 32.80% | 1.29 | 858702 | 3 | 22.38% | 3.10 | 1128583.3 | 3 | 21.80% | 4.08 |
| 105 | 64173.7 | 3 | 32.30% | 86790.3 | 3 | 22.30% | 1.35 | 72666.6 | 3 | 32.13% | 1.13 | 74025.6 | 3 | 22.30% | 1.15 |
| 106 | 52346.2 | 3 | 29.70% | 64657.3 | 3 | 23.29% | 1.24 | 64530.9 | 3 | 34.70% | 1.23 | 55498.1 | 3 | 31.70% | 1.06 |
| 107 | 57963.2 | 3 | 22.90% | 84657.3 | 3 | 28.20% | 1.46 | 193530.9 | 3 | 20.70% | 3.34 | 344988.1 | 3 | 22.90% | 5.95 |
| 108 | 1899668.5 | 3 | 26.20% | 2878769.5 | 3 | 22.24% | 1.52 | 2760131.8 | 3 | 22.20% | 1.45 | 3503997.3 | 3 | 22.20% | 1.84 |
| **Spot no** | **Control**  **(a)** | **N**  **(b)** | **CV**  **(c)** | **3 dpi**  **(a)** | **N**  **(b)** | **CV**  **(c)** | **Ratio**  **(d)** | **7 dpi**  **(a)** | **N**  **(b)** | **CV**  **(c)** | **Ratio**  **(d)** | **14 dpi**  **(a)** | **N**  **(b)** | **CV**  **(c)** | **Ratio**  **(d)** |
| 109 | 100700.1 | 3 | 31.70% | 160700.1 | 3 | 36.77% | 1.60 | 262744.1 | 3 | 22.80% | 2.61 | 204943.2 | 3 | 19.30% | 2.04 |

1. The average spot intensity of all gels in one replicate group after normalization by local regression model. The values are calculated based on the Gaussian distribution of the spots in the Gaussian 2-D image.
2. Number of replicate gels in a group.
3. Normalized coefficient of variation of the spot intensities within a replicate group.
4. The fold-change, i.e., the ratio of the average intensity of spots in the infected gels to the average intensity of matched spots in the control gels.
