# Supplementary material for: Proteomics approach combined with biochemical attributes to elucidate compatible and incompatible plant-virus interactions between Vigna mungo and Mungbean Yellow Mosaic India Virus
Source: Proteome Sci. 2013 Apr 15;11:15. doi: 10.1186/1477-5956-11-15 (PMC3639080; doi:10.1186/1477-5956-11-15)
Supplement: Additional file 6 — The leaf proteins identified by MALDI-MS and MS/MS analysis and categorized using Uniport database. [file 1477-5956-11-15-S6.doc]

**Table S2:** The leaf proteins identified by MALDI MS and MS/MS analysis and categorized using Uniport database. Category of unclassified proteins was modified according to their closest homologous protein obtained through BLAST in Uniport database.

| **Spot no.** | **Protein name** | **Species** | **Acce.**  **no. ^d^** | **Score ^e^** | **T**  **Mr / pI** | **PM ^f^** | **Peptide mass (observed) / Sequence of the peptide(s) matched by MS/MS** | **Sequence coverage**  **(MS / MS-MS)** | **Subcellular localization (TargetP)** |
| --- | --- | --- | --- | --- | --- | --- | --- | --- | --- |
| **Functional category: Energy** | | | | | | | | |  |
| 1 | Photosystem II oxygen-evolving complex protein 1^a^ | *Solanum tuberosum* | S16586 | 199 | 35.46 / 5.87 | 7 | 1760.8885/K.DGIDYAAVTVQLPGGER.V | 27 / 5 | Chloroplast |
| 2 | Ribulose-1,5-bisphosphate carboxylase small subunit ^a^ | *Vigna radiata* | Q9XQB5_PHAAU | 338 | 20.53 / 8.87 | 10 | 1702.6865/R.SGWIPCLEFELSHK.D  1996.8968/K.LPMYGCTDSSQVLKELR.E | 50 / 17 | Chloroplast |
| 3 | Photosystem II oxygen-evolving complex protein 1 precursor ^a^ | *Solanum tuberosum* | T06368 | 220 | 35.15 / 5.91 | 11 | 1760.9754/K.DGIDYAAVTVQLPGGER.V | 42 / 5 | Chloroplast |
| 5 | Rubisco activase ^a^ | *Vigna radiata* | AAD20019 | 269 | 48.04 / 7.57 | 10 | 940.4927 / K.FYWAPTR.D 1709.9572 / K.GLAYDISDDQQDITR.G  2062.2037/K.LLQYGNMLVQEQENVKR.V | 27 / 8 | Chloroplast |
| 8 | Chloroplast photosystem I reaction center subunit II-like protein ^a^ | *Oryza sativa* | Q84PB4_ORYSA | 203 | 22.13 / 9.72 | 7 | 1003.4545 / K.INYQFYR.V  1527. 6948 / R.VFPNGEVQYLHPK.D | 26 / 9 | Chloroplast |
| 9 | RbcL protein ^a^ | *Vigna radiata* | Q36849_PHAAU | 394 | 53.08 / 6.13 | 28 | 1465.8711/ K.TFQGPPHGIQVER.D | 46 / 2 | -  Contd. |
| **Spot no.** | **Protein name** | **Species** | **Acce.**  **no. ^d^** | **Score ^e^** | **T**  **Mr / pI** | **PM ^f^** | **Peptide mass (observed) / Sequence of the peptide(s) matched by MS/MS** | **Sequence coverage**  **(MS / MS-MS)** | **Subcellular localization (TargetP)** |
| 11 | Putative photosystem I reaction centre PSI-D subunit precursor ^a^ | *Solanum tuberosum* | Q70PN9_SOLTU | 162 | 22.85 / 9.63 | 8 | 1003.4585 / K.INYQFYR.V | 29 / 3 | Chloroplast |
| 12 | Photosystem I reaction center subunit IV B ^b^ | *Nicotiana sylvestris* | PSAEB_NICSY | 140 | 15.22 / 9.74 | 6 | 732.4430 / R.YPVVVR.F | 20 / 4 | Chloroplast |
| 13 | Rubisco activase ^a^ | *Vigna radiata* | AAD20019 | 249 | 48.04 / 7.57 | 14 | 1709.7461 / K.GLAYDISDDQQDITR.G | 33 / 3 | Chloroplast |
| 14 | Rubisco activase ^a^ | *Vigna radiata* | AAD20020 | 237 | 48.04 / 7.57 | 10 | 940.4116 / K.FYWAPTR.D  1709.7461 / K.GLAYDISDDQQDITR.G | 26 / 5 | Chloroplast |
| 18 | Ribulose-1,5-bisphosphate carboxylase small subunit ^a^ | *Vigna radiata* | Q9XQB5_PHAAU | 193 | 20.53 / 8.87 | 10 | 914.4199 / R.SPGYYDGR.Y | 42 / 4 | Chloroplast |
| 19 | Triose-phosphate isomerase ^a^ | *Phaseolus vulgaris* | Q5JZZ3_PHAVU | 205 | 27.41 / 5.87 | 7 | 1388.7144 / K.VIACIGETLEQR.E | 26 / 4 | - |
| 20 | Ribulose-1,5-bisphosphate carboxylase small subunit ^a^ | *Vigna radiata* | Q9XQB5_PHAAU | 190 | 20.53 / 8.87 | 9 | 1997.0529 / K.LPMYGCTDSSQVLKELR.E | 34 / 9 | Chloroplast |
| 21 | ATP synthase beta subunit ^a^ | *Nepenthes alata* | Q9MU27_NEPAL | 314 | 52.25 / 5.04 | 18 | 1328.6605 / K.AHGGVSVFGGVGER.T | 34 / 2 | - |
| 24 | Malate dehydrogenase (EC 1.1.1.37) precursor, NAD-dependent ^a^ | *Arabidopsis thaliana* | T49932 | 286 | 37.69 / 8.14 | 17 | 852.4459 / K.FADACLR.G | 35 / 1 | -  Contd. |
| **Spot no.** | **Protein name** | **Species** | **Acce. no. ^d^** | **Score ^e^** | **T**  **Mr / pI** | **PM ^f^** | **Peptide mass (observed) / Sequence of the peptide(s) matched by MS/MS** | **Sequence coverage**  **(MS / MS-MS)** | **Subcellular localization (TargetP)** |
| 29 | Chlorophyll a-b binding protein 4 ^b^ | *Solanum lycopersicum* | CB24_SOLLC | 128 | 28.98 / 5.33 | 7 | 1352.6085 / K.VGNFGEGRITMR.R | 12 / 4 | Chloroplast |
| 30 | Oxygen-evolving enhancer protein 2 ^b^ | *Solanum lycopersicum* | PSBP_SOLLC | 266 | 27.95 / 8.26 | 5 | 1572.7854 / K.SITDYGSPEEFLSK.V 1616.8042 / K.ENTDFLPYNGDGFK.L | 15 /10 | Chloroplast |
| 31 | Oxygen-evolving enhancer protein 3-2 ^b^ | *Arabidopsis thaliana* | PSBQ2_ARATH | 228 | 24.63 / 9.72 | 8 | 1261.6146 / K.AWPYVQNDLR.L 1389.7191/ K.KAWPYVQNDLR.L | 34 / 4 | Chloroplast |
| 32 | Glyceraldehyde-3-phosphate dehydrogenase (NADP) (phosphorylating) (EC 1.2.1.13) A ^a^ | *Spinacia oleracea* | DESPGA | 547 | 36.55 / 6.66 | 15 | 1571.6981 / K.GTMTTTHSYTGDQR.L  1382.7686 / K.KTFAEEVNAAFR.E 1254.6640 / K.TFAEEVNAAFR.E 1786.8903 / K.VIAWYDNEWGYSQR.V | 32 / 11 | Mitochondrion |
| 33 | Enolase (EC 2.3.1.16) ^a^ | *Glycine max* | Q6RIB7_SOYBN | 467 | 47.98 / 5.31 | 15 | 1804.9553 / R.AAVPSGASTGIYEALELR.D 1601.8055 / K.VNQIGSVTESIEAVR.M  1838.9156 / R.IEEELGAEAVYAGANFR.T | 31 / 11 | - |
| 36 | Plastoquinol-plastocyanin reductase (EC 1.10.99.1) Rieske iron-sulfur protein precursor ^a^ | *Pisum sativum* | S26199 | 297 | 24.68 / 8.63 | 9 | 1838.7955 / K.FICPCHGSQYNDQGR.V 1493.7993 / K.VVFVPWVETDFR.T | 33 / 11 | Chloroplast  Contd. |
| **Spot no.** | **Protein name** | **Species** | **Acce.**  **no. ^d^** | **Score ^e^** | **T**  **Mr / pI** | **PM ^f^** | **Peptide mass (observed) / Sequence of the peptide(s) matched by MS/MS** | **Sequence coverage**  **(MS / MS-MS)** | **Subcellular localization (TargetP)** |
| 37 | Probable fructose-bisphosphate aldolase (EC 4.1.2.13) precursor ^a^ | *Solanum tuberosum* | T07418 | 547 | 38.62 / 5.89 | 13 | 1387.7307 / R.LASIGLENTEANR.Q 1649.9720 / K.IVDVLIEQNIVPGIK.V 1114.5277 / R.SAAYYQQGAR.F 1452.8159 / R.TVVSIPNGPSALAVK.E | 34 / 14 | - |
| 38 | Putative mitochondrial NAD-dependent malate dehydrogenase ^a^ | *Solanum tuberosum* | Q8L5C8_SOLTU | 554 | 36.43 / 8.48 | 10 | 939.5348 / K.AGTYDEKR.L 1219.7228 / R.LFGVTTLDVVR.A 1318.6667 / R.DDLFNINAGIVK.S 1389.6786 / K.RTQDGGTEVVEAK.A 1576.8732 / K.ALEGADVVIIPAGVPR.K | 25 / 17 | Mitochondrion |
| 41 | Triose phosphate isomerase ^a^ | *Stellaria longipes* | AAB30759 | 275 | 27.71 / 5.54 | 10 | 954.4663 / K.FFVGGNWK.C 1285.5994 / K.TFDVCFKQLK.S | 25 / 7 | - |
| 53 | ATP synthase epsilon subunit^a^ | *Montinia caryophyllacea* | Q8M9D4_MONCA | 300 | 14.53 / 5.41 | 8 | 975.5911 / R.IVWDSEVK.E 1101.7345 / R.TRVEAINVIS.- 1284.8458 / K.RQTIEANLALR.R | 65 / 21 | - |
| 56 | Photosystem II oxygen-evolving complex protein 2 precursor ^a^ | *Solanum lycopersicum* | F2TOX2 | 299 | 27.95 / 8.26 | 8 | 1572.7607 / K.SITDYGSPEEFLSK.V 1616.7673 / K.ENTDFLPYNGDGFK.L | 27 / 10 | Chloroplast |
| 58 | Glyceraldehyde-3-phosphate dehydrogenase A subunit ^a^ | *Glycine max* | Q38IX1_SOYBN | 422 | 43.48 / 8.42 | 15 | 939.4644 / R.NPANLPWK.D 1254.6541 / K.TFAEEVNAAFR.E 1555.7172 / K.GTMTTTHSYTGDQR.L | 33 / 8 | Chloroplast  Contd. |
| **Spot no.** | **Protein name** | **Species** | **Acce. no. ^d^** | **Score ^e^** | **T**  **Mr / pI** | **PM ^f^** | **Peptide mass (observed) / Sequence of the peptide(s) matched by MS/MS** | **Sequence coverage**  **(MS / MS-MS)** | **Subcellular localization (TargetP)** |
| 60 | Glyceraldehyde-3-phosphate dehydrogenase A subunit ^a^ | *Glycine max* | Q38IX1_SOYBN | 320 | 43.48 / 8.42 | 13 | 939.4769 / R.NPANLPWK.D 1254.6690 / K.TFAEEVNAAFR.E | 27 / 4 | Chloroplast |
| 62 | Rubisco activase ^a^ | *Vigna radiata* | AAD20020 | 523 | 48.04 / 7.57 | 23 | 1623.7581 / K.MCALFINDLDAGAGR.L 1882.9656 / K.LVDTFPGQSIDFFGALR.A 1905.9638 / K.LLQYGNMLVQEQENVK.R 2089.1564 / R.VPIIVTGNDFSTLYAPLIR.D | 47 / 15 | Chloroplast |
| 66 | Ribulose-bisphosphate carboxylase (EC 4.1.1.39) small chain ^a^ | *Mesembryanthemum crystallinum* | S35246 | 168 | 20.3 / 7.63 | 8 | 913.4642 / R.YWTMWK.L 930.4890 / R.YWTFWK.L | 38 / 4 | Chloroplast |
| 67 | Rubisco activase ^a^ | *Vigna radiata* | AAD20020 | 496 | 48.04 / 7.57 | 17 | 940.4891 / K.FYWAPTR.D 1023.5114 / R.VYDDEVRK.W 1623.7581 / K.MCALFINDLDAGAGR.L 1709.8322 / K.GLAYDISDDQQDITR.G | 35 / 10 | Chloroplast |
| 68 | Rubisco activase ^a^ | *Vigna radiata* | AAD20020 | 312 | 48.04 / 7.57 | 14 | 940.4891 / K.FYWAPTR.D 1023.5114 / R.VYDDEVRK.W 1623.7581 / K.MCALFINDLDAGAGR.L | 34 / 6 | Chloroplast  Contd. |
| **Spot no.** | **Protein name** | **Species** | **Acce. no. ^d^** | **Score ^e^** | **T**  **Mr / pI** | **PM ^f^** | **Peptide mass (observed) / Sequence of the peptide(s) matched by MS/MS** | **Sequence coverage**  **(MS / MS-MS)** | **Subcellular localization (TargetP)** |
| 69 | Cytosolic malate dehydrogenase (EC 2.3.1.16) ^a^ | *Glycine max* | Q6RIB6_SOYBN | 416 | 35.85 / 6.32 | 11 | 749.3588 / R.KEGMER.K 1017.4866 / K.EFAPSIPEK.N 1757.8917 / R.KLSSALSAASAACDHIR.D 1629.8275 / K.LSSALSAASAACDHIR.D | 26 / 9 | - |
| 72 | Triosephosphate isomerase, putative ^c^ | *Ricinus communis* | gi|255576721 | 294 | 34.09 / 6.61 | 8 | 1285.6105 / K.TFDVCFQQLK.A 1646.9552 / K.VATPQQAQEVHVALR.D | 23 / 7 | Chloroplast |
| 73 | Rubisco activase ^a^ | *Vigna radiata* | AAD20019 | 303 | 48.04 / 7.57 | 13 | 1623.7581 / K.MCALFINDLDAGAGR.L 2089.1323 / R.VPIIVTGNDFSTLYAPLIR.D | 33 / 7 | Chloroplast |
| 76 | Putative ATP synthase beta subunit ^a^ | *Oryza sativa* | Q5N7P9_ORYSA | 574 | 45.94 / 5.33 | 14 | 1173.6578 / K.VVDLLAPYQR.G 1390.6352 / K.AHGGFSVFAGVGER.T 1492.7654 / R.FTQANSEVSALLGR.I 1662.7655 / K.CALVYGQMNEPPGAR.A | 32 / 12 | - |
| 77 | Pyruvate dehydrogenase (lipoamide) (EC 1.2.4.1) beta chain ^a^ | *Pisum sativum* | T06532 | 207 | 38.4 / 5.88 | 10 | 1200.6627 / K.EGISAEVINLR.S 1768.9084 / K.SNYMSAGQISVPIVFR.G | 20 / 7 | Mitochondrion |
| 84 | Putative mitochondrial NAD-dependent malate dehydrogenase ^a^ | *Solanum tuberosum* | Q8L5C8_SOLTU | 227 | 36.43 / 8.48 | 7 | 1219.7442 / R.LFGVTTLDVVR.A 1576.9252 / K.ALEGADVVIIPAGVPR.K | 23 / 7 | Mitochondrion  Contd. |
| **Spot no.** | **Protein name** | **Species** | **Acce. no. ^d^** | **Score ^e^** | **T**  **Mr / pI** | **PM ^f^** | **Peptide mass (observed) / Sequence of the peptide(s) matched by MS/MS** | **Sequence coverage**  **(MS / MS-MS)** | **Subcellular localization (TargetP)** |
| 85 | Ferredoxin:NADP+ reductase (EC 1.18.1.2), chain A ^a^ | *Pisum sativum* | 1QG0A | 356 | 33.8 / 6.2 | 10 | 1265.6267 / K.KAEQWNVEVY.- 1347.6274 / K.DNTFVYMCGLK.G 1630.8136 / R.LYSIASSAIGDFGDSK.T | 36 / 12 | - |
| 88 | Ribulose-bisphosphate carboxylase (EC 4.1.1.39) precursor small chain ^a^ | *Solanum tuberosum* | RKPO2C | 153 | 20.53 / 7.6 | 6 | 914.3957 / K.SPGYYDGR.Y | 36 / 4 | Chloroplast |
| 92 | Unknown ^c^ | *Glycine max* | gi|255635100 | 234 | 33.39 / 6.35 | 8 | 954.4908 / K.FFVGGNWK.C 1082.5709 / K.WVVLGHSER.R | 29 / 5 | Chloroplast |
| 94 | ATP synthase CF1 epsilon subunit ^a^ | *Glycine max* | Q2PMU9_SOYBN | 304 | 14.8 / 5.41 | 6 | 975.5004 / R.IVWDSEVK.E 1128.5973 / R.QTIEANLALR.R 1685.7943 / R.INNNEITVLVNDAEK.G | 43 / 24 | - |
| 102 | Cytochrome c oxidase subunit 6b-1^a^ | *Oryza sativa* | Q9SXV0_ORYSA | 216 | 19.13 / 4.27 | 5 | 1019.5427 / K.IETAPADFR.F 1232.5887 / R.SLCPGEWVER.W | 25 / 11 | - |
| 103 | Rubisco activase ^a^ | *vigna radiata* | AAD20019 | 293 | 48.04 / 7.57 | 13 | 1023.5114 / R.VYDDEVRK.W 1228.6076 / K.SFQCELVFAK.M 1709.7679 / K.GLAYDISDDQQDITR.G | 23 / 7 | Chloroplast |
| **Functional category: Metabolism** | | | | | | | | |  |
| 10 | Glutamine synthetase ^b^ | *Phaseolus vulgaris* | GLNA4_PHAVU | 223 | 47.5 / 6.77 | 8 | 1626. 7092 / R.HKEHISAYGEGNER.R | 14 / 3 | Chloroplast |
| 15 | Phosphoribulokinase ^a^ | *Pisum sativum* | P93681_PEA | 171 | 39.23 / 5.41 | 10 | 1343.5990 / K.FYGEVTQQMLK.H | 25 / 3 | -  Contd. |
| **Spot no.** | **Protein name** | **Species** | **Acce. no.^d^** | **Score ^e^** | **T**  **Mr / pI** | **PM ^f^** | **Peptide mass (observed) / Sequence of the peptide(s) matched by MS/MS** | **Sequence coverage**  **(MS / MS-MS)** | **Subcellular localization (TargetP)** |
| 22 | Glycine hydroxymethyltransferase (EC 2.1.2.1) isoform 2^a^ | *Flaveria pringlei* | S40213 | 287 | 57.07 / 8.8 | 20 | 999.4807/ K.YSEGYPGAR.Y  1259.6645/ K.LRHDVEEYAK.Q | 30 / 3 | Mitochondrion |
| 23 | Glycine hydroxymethyltransferase (EC 2.1.2.1) isoform 2^a^ | *Flaveria pringlei* | S40213 | 231 | 57.07 / 8.8 | 20 | 999.4807 / K.YSEGYPGAR | 30 / 1 | Mitochondrion |
| 27 | Aminomethyltransferase (EC 2.1.2.10) precursor ^a^ | *Pisum sativum* | S56661 | 368 | 44.66 / 8.79 | 20 | 1407.6477 / K.GGDVSWHIHDER.S | 36 / 2 | Mitochondrion |
| 28 | Aminomethyltransferase (EC 2.1.2.10) precursor ^a^ | *Pisum sativum* | S56662 | 414 | 44.66 / 8.79 | 12 | 1113.6414/ R.VGFISSGPPPR.S 1269.7257/ R.RVGFISSGPPPR.S 1407.6833/ K.GGDVSWHIHDER.S | 25 / 5 | Mitochondrion |
| 43 | Sedoheptulose-bisphosphatase (EC 3.1.3.37) precursor ^a^ | *Arabidopsis thaliana* | S51838 | 327 | 42.82 / 6.47 | 15 | 1633.8856 / R.YTGGMVPDVNQIIVK.E 1649.8854 / R.YTGGMVPDVNQIIVK.E | 32 / 7 | Chloroplast |
| 44 | Glycine cleavage system protein H precursor ^a^ | *Flaveria pringlei* | S60194 | 144 | 17.46 / 5.04 | 5 | 1123.4063 / K.FCEEEDSAH.- | 27 / 5 | Mitochondrion |
| 45 | Glycine hydroxymethyltransferase (EC 2.1.2.1) ^a^ | *Solanum tuberosum* | S40218 | 441 | 57.22 / 8.4 | 13 | 999.4366 / K.YSEGYPGAR.Y 1569.7584 / K.NTVPGDVSAMVPGGIR.M 1259.5873 / K.LRHDVEEYAK.Q | 24 / 6 | Mitochondrion |
| 48 | Putative ribose 5-phosphate isomerase ^a^ | *Arabidopsis thaliana* | Q8L9K5_ARATH | 284 | 29.42 / 5.73 | 7 | 889.4407 / K.RTEEQAR.S 2025.0435 / R.IDLAIDGADEVDPNLDLVK.G | 15 / 9 | Chloroplast  Contd. |
| **Spot no.** | **Protein name** | **Species** | **Acce. no. ^d^** | **Score ^e^** | **T**  **Mr / pI** | **PM ^f^** | **Peptide mass (observed) / Sequence of the peptide(s) matched by MS/MS** | **Sequence coverage**  **(MS / MS-MS)** | **Subcellular localization (TargetP)** |
| 52 | Similarity to 30S ribosomal protein S10 ^a^ | *Arabidopsis thaliana* | Q9LK61_ARATH | 299 | 20.99 / 8.99 | 10 | 848.4660 / R.FHFEIR.T 1496.7379 / R.SYWVPLIEDSCK.Q | 27 / 9 | Chloroplast |
| 57 | AlaT1^a^ | *Vitis vinifera* | Q45RS3_VITVI | 341 | 54.06 / 6.54 | 13 | 1257.7085 / K.GVMQILNTIIR.G 1783.9008 / K.HYLSLTSGGLGAYSDSR.G | 22 / 5 | - |
| 74 | Chlorophyll magnesium chelatase (EC 4.99.1.-) - soybean chloroplast ^a^ | *Glycine max* | JC4312 | 448 | 46.07 / 5.49 | 13 | 945.5390 / K.AFEPGLLAK.A 1202.6139 / R.FGMHAQVGTVR.D 1245.6286 / K.VCAELNVDGLR.G 1543.7769 / K.INMVDLPLGATEDR.V | 29 / 10 | Chloroplast |
| 75 | Chlorophyll magnesium chelatase (EC 4.99.1.-) - soybean chloroplast ^a^ | *Glycine max* | JC4312 | 531 | 46.07 / 5.49 | 17 | 1048.5665 / K.IGGVMIMGDR.G 1202.6139 / R.FGMHAQVGTVR.D 1245.6286 / K.VCAELNVDGLR.G  1543.7443 / K.INMVDLPLGATEDR.V | 41 / 10 | Chloroplast |
| 81 | N-glyceraldehyde-2 phosphotransferase ^a^ | *Arabidopsis thaliana* | Q9LHT3_ARATH | 207 | 31.99 / 5.14 | 8 | 1123.6185 / K.IQYGTLCIR.E 1391.6896 / R.ENPGCLFIATNR.D | 23 / 7 | - |
| 86 | Gamma hydroxybutyrate dehydrogenase-like protein ^a^ | *Oryza sativa* | Q84VC8_ORYSA | 132 | 30.65 / 6.18 | 6 | 1624.8448 / K.KPAEDGQLVILAAGDK.V | 22 / | - |
| 87 | Ribosomal protein S3 ^c^ | *Cuscuta exaltata* | gi|159161288 | 123 | 25.36 / 9.97 | 7 | 869.4690 / R.VPRQTIR.A | 24 / 3 | -  Contd. |
| **Spot no.** | **Protein name** | **Species** | **Acce. no. ^d^** | **Score ^e^** | **T**  **Mr / pI** | **PM ^f^** | **Peptide mass (observed) / Sequence of the peptide(s) matched by MS/MS** | **Sequence coverage**  **(MS / MS-MS)** | **Subcellular localization (TargetP)** |
| 89 | Putative ribose-5-phosphate isomerase ^a^ | *Oryza sativa* | Q6ZEZ2_ORYSA | 136 | 29.35 / 5.52 | 6 | 921.5449 / -.MAAATVSVR.F | 19 / 3 | Chloroplast |
| 91 | Hydroxyethylthiazole kinase-like protein ^a^ | *Oryza sativa* | Q5ZDB4_ORYSA | 134 | 23.69 / 8.24 | 7 | 1271.6486 / R.AWELLSAVRAR.A | 29 / 5 | Mitochondrion |
| 96 | Nucleoside diphosphate kinase (EC 2.7.4.6) ^a^ | *Glycine max* | Q8GV25_SOYBN | 191 | 16.4 / 6.91 | 7 | 1624.8583 / K.IIGATNPAQSEPGTIR.G | 40 / 10 | - |
| 97 | Aminomethyltransferase (EC 2.1.2.10) precursor ^a^ | *Pisum sativum* | S56661 | 282 | 44.66 / 8.79 | 13 | 1113.6739 / R.VGFISSGPPPR.S 1407.6601 / K.GGDVSWHIHDER.S | 26 / 5 | Mitochondrion |
| 98 | Phosphoribulokinase (EC 2.7.1.19) ^a^ | *Pisum sativum* | T06463 | 225 | 39.23 / 5.41 | 9 | 1450.6275 / R.KPDFEAYIDPQK.Q 1642.8064 / K.ILVIEGLHPMYDSR.V | 34 / 7 | - |
| 100 | Aldehyde reductase ^a^ | *Vigna radiata* | Q9SQK0_9FABA | 343 | 35.79 / 6.33 | 8 | 792.5262 / R.YLLVER.V 915.4549 / R.VAHFGDAAK.I 1546.8064 / K.SLGIDYIPLEVSLK.D 1761.0115 / R.VVLTSSIAAVAFSDRPK.N | 26 / 14 | Secretory pathway |
| 105 | Probable gamma-glutamyl hydrolase ^a^ | *Glycine max* | T08837 | 271 | 37.82 / 6.08 | 8 | 2157.0412 / K.NAFEWATSLKAPHTEDAIR.V 2239.1420 / R.NDAGDHFPVIAFNLGGNLVIR.I | 22 / 11 | Secretory pathway |
| 106 | Coproporphyrinogen-III oxidase, chloroplastic ^b^ | *Hordeum vulgare* | HEM6_HORVU | 286 | 43.99 / 8.05 | 12 | 1317.6519 / R.YVEFNLVYDR.G 1411.8488 / R.IESILVSLPLTAR.W 1476.7393 / K.NPFAPTLHFNYR.Y | 27 / 8 | Chloroplast  Contd. |
| **Spot no.** | **Protein name** | **Species** | **Acce. no. ^d^** | **Score ^e^** | **T**  **Mr / pI** | **PM ^f^** | **Peptide mass (observed) / Sequence of the peptide(s) matched by MS/MS** | **Sequence coverage**  **(MS / MS-MS)** | **Subcellular localization (TargetP)** |
| 107 | Translation elongation factor EF-Tu precursor, chloroplast ^a^ | *Glycine max* | S60659 | 312 | 52.18 / 6.21 | 11 | 1681.9521 / K.ILDEALAGDNVGLLLR.G 1768.7845 / R.HYAHVDCPGHADYVK.N 1810.8928 / R.GITINTATVEYETENR.H | 21 / 9 | Chloroplast |
| **Functional category: Stress/defense** | | | | | | | | |  |
| 4 | Iron-superoxide dismutase ^a^ | *Glycine max* | Q71UA1_SOYBN | 275 | 27.51 / 5.45 | 7 | 1826.8833 / K.AAAATQFGSGWAWLAYR.A | 27 / 6 | - |
| 6 | Stromal 70 kDa heat shock-related protein ^b^ | *Pisum sativum* | HSP7S_  PEA | 276 | 75.58 / 5.22 | 13 | 1751.7798 / R.IINEPTAASLAYGFER.K  1566.7140 / K.AVVTVPAYFNDSQR.T | 19 / 4 | Chloroplast |
| 7 | Chaperonin 60 alpha chain precursor ^a^ | *Brassica napus* | S38642 | 244 | 61.68 / 5.14 | 16 | 1754.8750 / R.AIELPDAMENAGAALIR.E | 21 / 2 | Chloroplast |
| 25 | Putative ankyrin-repeat protein ^a^ | *Vitis aestivalis* | Q6TKQ6_VITAE | 253 | 38.09 / 4.53 | 9 | 1293.6536 / K.NTALHYAAGYGR.K 1452.7135/ R.TALHFACGYGEVK.C | 20 / 7 | Chloroplast |
| 26 | Catalase (EC 1.11.1.6) ^a^ | *Vigna radiata* | T10902 | 473 | 38.09 / 8.14 | 22 | 988.5327 / R.FSTVIHER.G 1255.6033/ R.DEEVNYFPSR.Y | 42 / 3 | - |
| 34 | Cytosolic ascorbate peroxidase ^a^ | *Vigna unguiculata* | Q41712_VIGUN | 356 | 27.07 / 5.64 | 15 | 1258.5960 / K.SYPTVSADYQK.A 1854.9061 / K.HPAELAHGANNGLDIAVR.L | 66 /11 | - |
| 39 | Unknown protein ^c^ | *Glycine max* | gi |255645102 | 358 | 26.64 / 6.77 | 9 | 1133.6709 / K.YTAIKPLGDR.V 1603.8920 / R.KPLSVTPGNTVLYSK.Y | 34 / 9 | Chloroplast  Contd. |
| **Spot no.** | **Protein name** | **Species** | **Acce. no. ^d^** | **Score ^e^** | **T**  **Mr / pI** | **PM ^f^** | **Peptide mass (observed) / Sequence of the peptide(s) matched by MS/MS** | **Sequence coverage**  **(MS / MS-MS)** | **Subcellular localization (TargetP)** |
| 40 | Cytosolic ascorbate peroxidase ^a^ | *Vigna unguiculata* | Q41712_VIGUN | 409 | 27.07 / 5.64 | 11 | 1250.6121 / R.EDKPEPPPEGR.L 1854.9745 / K.HPAELAHGANNGLDIAVR.L 2031.9489 / K.YAADEDAFFADYAVAHQK.L | 54 / 18 | - |
| 42 | Peptidyl-prolyl cis-trans isomerase ^b^ | *Spinacia oleracea* | TLP40_SPIOL | 330 | 50.07 / 5.29 | 12 | 933.5032 / R.YALPIDNK.A 1295.6018 / R.HFYDGMEIQR.R 1426.7041 /K.LPFNAFGTMAMAR.E | 15 / 6 | Chloroplast |
| 46 | Probable chaperonin 60 beta chain ^a^ | *Pisum sativum* | T06412 | 401 | 63.29 / 5.85 | 15 | 733.3921 / K.APGFGER.K 1280.7860 / K.VVAAGANPVLITR.G 1505.7140 / R.GYISPYFVTDSEK.M 1700.8967 / K.AAVEEGIVVGGGCTLLR.L | 28 / 8 | Chloroplast |
| 49 | Putative CuZn-superoxide dismutase (EC 1.15.1.1) ^a^ | *Populus tremula x Populus tremuloides* | Q9BA07_9ROSI | 280 | 21.59 / 6.23 | 6 | 1237.6204 / K.LTHGAPEDEIR.H 1365.7005 / K.KLTHGAPEDEIR.H | 26 / 5 | Chloroplast |
| 50 | Thioredoxin M ^a^ | *Zea mays* | T03957 | 139 | 18.46 / 8.7 | 6 | 1017.6685 / R.SIPTVLIFK.G | 27 / 5 | Chloroplast |
| 51 | Cytoplasmic  Cu/Zn SOD ^a^ | *Gossypium hirsutum* | Q3SAX1_GOSHI | 294 | 15.34 / 5.47 | 5 | 1153.5903 / K.EHGAPEDENR.H 1335.6824 / R.AVVVHADPDDLGK.G | 40 / 15 | - |
| 54 | Cyclophilin-like protein (EC 5.2.1.8) ^a^ | *Triticum aestivum* | Q6XPZ6_WHEAT | 355 | 26.06 / 9.4 | 13 | 1272.5542 / K.DFMIQGGDFDK.G 1698.8904 / R.HVVFGQVLEGMDIVR.T | 43 / 10 | Chloroplast  Contd. |
| **Spot no.** | **Protein name** | **Species** | **Acce. no. ^d^** | **Score ^e^** | **T**  **Mr / pI** | **PM ^f^** | **Peptide mass (observed) / Sequence of the peptide(s) matched by MS/MS** | **Sequence coverage**  **(MS / MS-MS)** | **Subcellular localization (TargetP)** |
| 55 | Peptidyl-prolyl cis-trans isomerase ^a^ | *Medicago truncatula* | Q1SQJ6_MEDTR | 220 | 28.22 / 7.66 | 10 | 901.4885 / K.TPWLDNR.H | 28 / 2 | Chloroplast |
| 65 | Quinone oxidoreductase-like protein At1g23740 ^a^ | *Arabidopsis thaliana* | E86371 | 314 | 32.81 / 5.65 | 7 | 1020.5012 / K.LNPYIESGK.V 1148.5806 / K.KLNPYIESGK.V 1266.6012 / R.SLGADLAIDYTK.E 1571.7841 / K.QFGSLAEYTAVEEK.L | 26 / 11 | Chloroplast |
| 71 | BTF3b-like transcription factor, putative ^c^ | *Arabidopsis thaliana* | gi|15220876 | 353 | 17.96 / 6.62 | 6 | 863.4531 / K.LAEQFQK.Q 1287.7059 / K.DDVVIQFINPK.V 1943.9899 / K.VQASIAANTWVVSGSPQTK.K | 35 / 22 | - |
| 79 | Dihydrolipoamide dehydrogenase ^a^ | *Vigna unguiculata* | T06332 | 369 | 53.31 / 6.90 | 15 | 1153.6428 / K.HIIIATGSDVK.S 1466.6773 / R.VCHAHPTMSEAVK.E 1523.7569 / K.ALLHSSHMYHEAK.H | 26 / 7 | Mitochondrion |
| 80 | Dihydrolipoamide dehydrogenase (EC 1.8.1.4) 2 precursor ^a^ | *Glycine max* | T06332 | 207 | 53.31 / 6.9 | 8 | 980.5315 / K.HAFANHGVK.F 1523.7431 / K.ALLHSSHMYHEAK.H | 17 / 4 | Mitochondrion |
| 101 | Uridylyltransferase-related ^c^ | *Arabidopsis thaliana* | gi|18394414 | 275 | 31.45 / 4.98 | 9 | 1283.6775 / K.VEDPELLEAIR.L 1411.7696 / R.KVEDPELLEAIR.L | 19 / 4 | Chloroplast |
| 109 | Type II peroxiredoxin ^c^ | *Populus trichocarpa* | gi|224126457 | 152 | 17.52 / 5.35 | 6 | 912.4808 / K.HVPGFVEK.A | 25 / 4 | -  Contd. |
| **Spot no.** | **Protein name** | **Species** | **Acce. No. ^d^** | **Score ^e^** | **T**  **Mr / pI** | **PM ^f^** | **Peptide mass (observed) / Sequence of the peptide(s) matched by MS/MS** | **Sequence coverage**  **(MS / MS-MS)** | **Subcellular localization (TargetP)** |
| **Functional category: Transport** | | | |  |  |  |  |  |  |
| 16 | Mitochrondrial voltage-dependent anion-selective channel ^a^ | *Phaseolus coccineus* | Q4PKP6_PHACN | 255 | 29.71 / 8.56 | 12 | 739.3732 / K.AIFNFK.V | 44 / 2 | - |
| 70 | Mitochrondrial voltage-dependent anion-selective channel ^a^ | *Phaseolus coccineus* | Q4PKP6_PHACN | 571 | 29.71 / 8.56 | 12 | 739.4105 / K.AIFNFK.V 1330.6235 / K.SFFTISGEVDTK.A 1945.9913 / K.FTVTTYSPTGVAITSSGTR.K 2515.3218 / R.FSTNENTITLGTQHALDPLTTLK.A  2853.4097 / K.GDALNAAYYHVVNPLTNTAVGAEVTHR.F | 55 / 31 | - |
| **Functional category: Secondary Metabolism** | | | | | | | | |  |
| 17 | Isoflavone reductase-like protein 5 ^a^ | *Vitis vinifera* | Q3KN68_VITVI | 276 | 33.87 / 5.76 | 15 | 1429.5113 / R.FFPSEFGNDVDR.V | 31 / 3 | - |
| 63 | Chavicol O-methyltransferase ^b^ | *Ocimum basilicum* | CVMT1_OCIBA | 207 | 40.23 / 5.67 | 8 | 973.5346 / K.LANKPSMGR.F 1319.6442 / R.TLVDVGGGNGTMAK.A | 27 / 6 | - |
| **Functional category: Signal Transduction** | | | | | | | | |  |
| 104 | Calmodulin ^a^ | *Spinacia oleracea* | MCSP | 307 | 16.78 / 4.15 | 8 | 956.5223/K.EAFSLFDK.D 1809.8863/R.VFDKDQNGFISAAELR.H 1927.8822/R.EADVDGDGQINYEEFVK.V | 48 / 27 | -  Contd. |
| **Spot no.** | **Protein name** | **Species** | **Acce. no. ^d^** | **Score ^e^** | **T**  **Mr / pI** | **PM ^f^** | **Peptide mass (observed) / Sequence of the peptide(s) matched by MS/MS** | **Sequence coverage**  **(MS / MS-MS)** | **Subcellular localization (TargetP)** |
| 108 | Protein phosphatase 2C ^a^ | *Hyacinthus orientalis* | Q676W9_HYAOR | 143 | 16.16 /7.11 | 6 | 1157.6299 / -.NLGDSRAVLGR.K | 28 / 7 | - |
| **Functional category: Transcription** | | | | | | | | |  |
| 59 | Putative mRNA binding protein ^a^ | *Oryza sativa* | Q8GTK8_ORYSA | 236 | 41.27 / 7.68 | 11 | 765.5046 / K.KAFPFR.N 1303.5327 / K.DCEEWFFDR.I | 26 / 3 | Chloroplast |
| 82 | 28 kDa ribonucleoprotein, chloroplastic ^b^ | *Spinacia oleracea* | ROC1_SPIOL | 178 | 25.33 / 4.4 | 6 | 1069.6639 / R.VNVAEERPR.R 1187.7185 / R.LEQLFSEHGK.V | 24 / 8 | - |
| 83 | RNA-binding protein ^a^ | *Mesembryanthemum crystallinum* | S50765 | 184 | 32.0 / 4.47 | 6 | 1069.6210 / R.VNVAEERPR.R 1173.6464 / R.LEQVFSEHGK.V | 20 / 6 | Chloroplast |
| **Functional category: Protein storage** | | | | | | | | |  |
| 64 | Vicilin^a^ | *Pisum sativum* | S08505 | 316 | 52.26 / 5.39 | 16 | 1196.6318 / K.IFENLQNYR.L 1385.7264 / K.EGSLLLPHYNSR.A | 33 / 4 | Secretory pathway |
| **Functional category: Unclassified** | | | | | | | | |  |
| 35 | IN2-2 protein ^b^ | *Zea mays* | IN22_MAIZE | 349 | 34.09 / 8.88 | 8 | 1367.7227 / K.YIGLSEASASTIR.R 1444.8301 / R.ELGIGIVAYSPLGR.G | 25 / 8 | - |
| 47 | PREDICTED: hypothetical protein ^c^ | *Vitis vinifera* | gi|225457361 | 177 | 30.83 / 6.31 | 7 | 1942.9436 / K.GVNPWIEVDGGVGPANAYK.V | 29 / 6 | Chloroplast |
| 61 | SHOOT1 protein ^a^ | *Glycine max* | Q9AT39_SOYBN | 346 | 40.5 / 5.26 | 12 | 1103.5889 / R.EIQMLNALR.K 1607.7295 / R.DGGTYIDAIAPGGSADK.A 1724.8230 / R.FDESFINENAINAIK.S | 35 /11 | Chloroplast  Contd. |
| **Spot no.** | **Protein name** | **Species** | **Acce. no. ^d^** | **Score ^e^** | **T**  **Mr / pI** | **PM ^f^** | **Peptide mass (observed) / Sequence of the peptide(s) matched by MS/MS** | **Sequence coverage**  **(MS / MS-MS)** | **Subcellular localization (TargetP)** |
| 78 | Pyridoxal-5-phosphate-dependent enzyme, beta subunit ^a^ | *Medicago truncatula* | Q1SUH8_MEDTR | 284 | 40.97 / 6.48 | 6 | 1320.7332 / K.LIGVVFPSFGER.Y 1365.5863 / K.LEIMEPCCSVK.D 1461.7647 / K.LILTMPASMSLER.R | 15 / 9 | Chloroplast |
| 90 | Haloacid dehalogenase-like hydrolase family protein ^a^ | *Arabidopsis thaliana* | gi|18420570 | 243 | 35.09 / 5.4 | 7 | 1131.5891 / K.LIDTLQDWK.T 1584.7042 / R.FQGLDCFLAGDDVK.E | 24 / 7 | Chloroplast |
| 93 | ENT domain-containing protein ^c^ | *Arabidopsis thaliana* | gi|15229958 | 123 | 36.89 / 5.74 | 7 | 1759.8967 / R.LVSSGISGNESAEALIGR.K | 18 / 5 | - |
| 95 | Glycine-rich RNA-binding protein RGP-1c ^a^ | *Nicotiana sylvestris* | S41773 | 253 | 16.7 / 5.29 | 7 | 1131.6258 / R.NITVNEAQSR.G 1577.7057 / R.GFGFVTFSNEDAMR.S | 44 / 14 | - |
| 99 | Unknown ^c^ | *Glycine max* | gi|255639881 | 280 | 12.27 / 9.98 | 7 | 1146.4897 / R.ILEEVDTAEK.A 2607.3796 / R.DMYSALNAVSGHYISFGPTAPIPAK.R | 63 / 31 | - |

^a^ Protein identified in MSDB database.

^b^ Protein identified in Swissprot database.

^c^ Protein identified in NCBInr database.

^d^ Accession number.

^e^ MS and MS/MS combined score.

^f^ Number of matched peptides.
