# Supplementary material for: Proteomics approach combined with biochemical attributes to elucidate compatible and incompatible plant-virus interactions between Vigna mungo and Mungbean Yellow Mosaic India Virus
Source: Proteome Sci. 2013 Apr 15;11:15. doi: 10.1186/1477-5956-11-15 (PMC3639080; doi:10.1186/1477-5956-11-15)
Supplement: Additional file 7 — Venn diagrams depicting the distribution of differentially abundant proteins at different time points. [file 1477-5956-11-15-S7.doc]

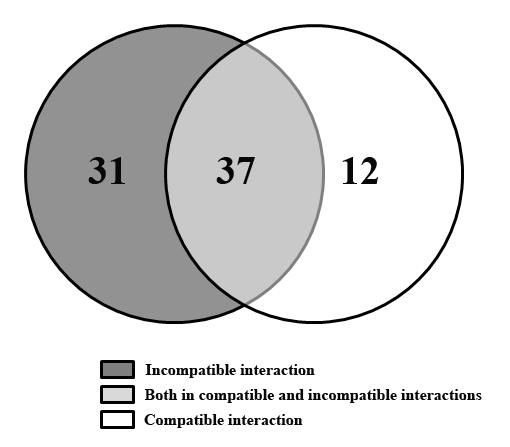


**Figure S5:** Venn diagrams depicting the distribution of differentially abundant proteins at three different time points after challenging with MYMIV.
