# Supplementary material for: Proteomics approach combined with biochemical attributes to elucidate compatible and incompatible plant-virus interactions between Vigna mungo and Mungbean Yellow Mosaic India Virus
Source: Proteome Sci. 2013 Apr 15;11:15. doi: 10.1186/1477-5956-11-15 (PMC3639080; doi:10.1186/1477-5956-11-15)
Supplement: Additional file 9 — Involvement of the identified proteins in the pentose phosphate pathway assigned by KEGG database in Blast2go software. [file 1477-5956-11-15-S9.doc]

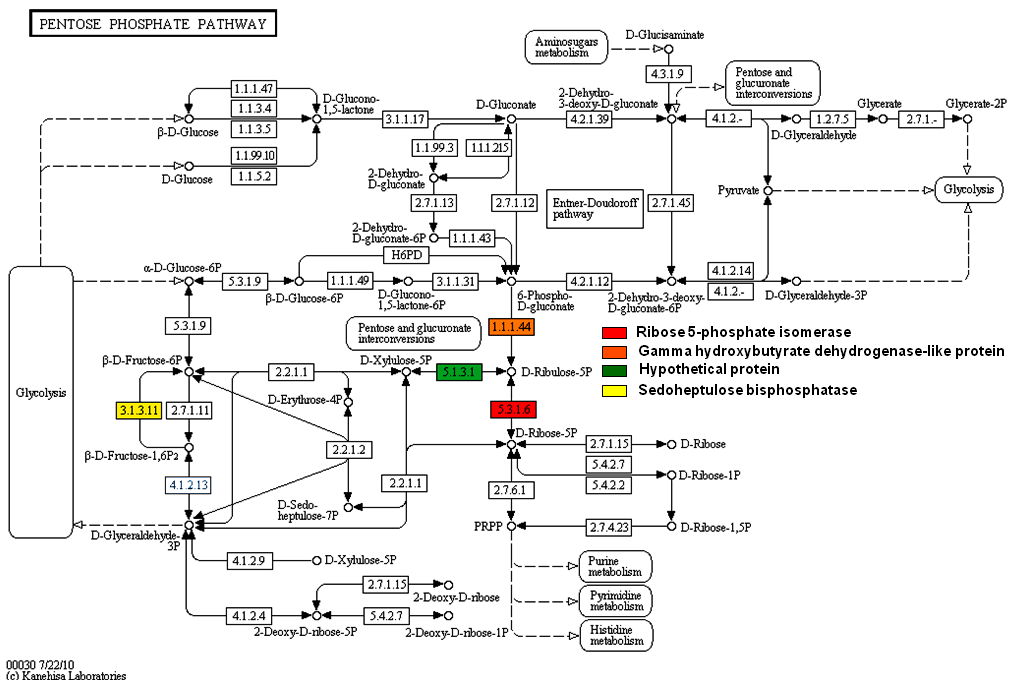


**Figure S7:** Involvement of the identified proteins in the pentose phosphate pathway assigned by KEGG database in Blast2go software.
